# Supplementary material for: Machine learning aided design of single-atom alloy catalysts for methane cracking
Source: Nat Commun. 2024 Jul 18;15:6036. doi: 10.1038/s41467-024-50417-7 (PMC11255339; doi:10.1038/s41467-024-50417-7)
Supplement: Supplementary file 1 — Supplementary Information [file 41467_2024_50417_MOESM1_ESM.pdf]

Supplementary Information for

Machine Learning Aided Design of Single-atom Alloy Catalysts for Methane Cracking

Jikai Sun[1,2], Rui Tu[1,2], Yuchun Xu[1], Hongyan Yang[1], Tie Yu[1,*], Dong Zhai[1], Xiuqin Ci[1], Weiqiao Deng[1,*]

Affiliations

[1]Institute of Frontier Chemistry, School of Chemistry and Chemical Engineering, Shandong University, Binhai Road No.72, 266237, Qingdao China

[2]These authors contributed equally

**Corresponding authors.*

Email address: yutie@sdu.edu.cn; dengwq@sdu.edu.cn

1. Supplementary Note

1.1 Activity test

The methane cracking reaction was studied by a self-made mechanical system, which used a motor to control the vibration frequency of the reactor. The system can support mechanical catalysis below 450°C and vibration frequency of 0-1500 r/min (the motor speed corresponding to the vibration frequency of ball milling reactor is about 0-25 Hz). Typically, 333 g metal balls are sealed in a reactor, and the temperature is controlled by a K-type thermocouple inserted into the reactor. The feed gas is pure CH_4 with a flow rate of 50 mL/min. The reaction is carried out at atmospheric pressure. The products were analyzed by on-line gas chromatography (ThermoFisher Trace 1300), including thermal conductivity detector (TCD) and flame ionization detector (FID). Porapak Q and 5 molecular sieve columns are connected to

TCD, while TG WAXMS and HP AL/S capillary columns are connected to FID. Hydrocarbons are analyzed by FID, while CO and CO_2 are analyzed by TCD.

1.2 Electrochemical tests method

Electrochemical tests of as-synthesized samples and commercial graphite were conducted using CR2032 coin cells with a glass fiber (GF/A, Whatman) and sodium metal sheets as counter electrodes. To obtain working electrodes, a slurry containing the active materials conductive, carbon black and polyvinylidene fluoride (PVDF) with a mass ratio of 7:2:1 in N-methylpyrrolidone (NMP) was coated on steel sheets and dried at 120 ℃ for 12 h. The electrolyte was 1 M $NaPF_6$ in ethylene carbonate/dimethyl carbonate/ethyl methyl carbonate (EC:DMC:EMC =1:1:1 in volume). Then, the cells were assembled in an argon-filled glove box with 70 μL of electrolyte. The mass loading of the active material was approximately 1 mg $cm^{-2.}$ Galvanostatic charging/discharging tests and cycling stability tests were carried out in a LAND CT3001A battery testing system with a voltage range of 0.01 - 3 V vs. Na/Na^+ at room temperature.

1.3 Surface model construction

The most stable bulk models of various transition metals were obtained from the Materials Project[1] database and optimized using density functional theory (DFT). All potential surfaces were then obtained by cutting these models. By replacing one host metal in the top surface layer with a doping atom, a total of 10,960 transition metal singel-atom alloys (SAAs) surface models were generated. For each surface model, the thickness of the vacuum layer was set to 15 Å. The lattice dimensions a and b were ensured to be larger than 6 Å by supercell expansion, and an appropriate number

of layers were chosen to ensure a thickness greater than 5 Å. During surface optimization, the top two layers were fully relaxed while the other underlying metal atoms were holding fixed at their equilibrium bulk phase position. As shown in Supplementary Fig. 2, all possible adsorption sites, such as top, bridge, hollow, etc., were considered to determine the most stable adsorption site for CH intermediates.

1.4 Calculation of reaction rates

The reaction rate (k) is calculated by the transition state theory (TST) theory:

$$k=\frac{k_B T}{h}\left(\frac{RT}{P_0}\right)^{\Delta n} e^{\frac{-E_b}{k_B T}} \qquad 1)$$

where k_B, h, and R are the Boltzmann constant, Planck constant, and molar gas constant, respectively. T is the reaction temperature, P_0 is the inverse of the molar concentration, and E_b is the energy barrier.

The total reaction rate (k_{tot}) of SAAs for all surfaces is calculated using the formula 2:

$$k_{tot}=\sum_{i=0}^{n} a_i \times k_i \qquad 2)$$

where a_i is the surface proportion and k_i is the reaction rate on this surface.

1.5 Machine learning

Feature Engineering

A process to automatically acquire and filter descriptors for SAAs was designed. As shown in Supplementary Table 1, descriptors were divided into three categories: descriptors describing the properties of the elements and the host surface, descriptors related to the coordination information of the substrate surface, new descriptors composed by combining the properties of the elements with the coordination number

based on expert knowledge. The descriptors describing the properties of the elements and the host surface were sourced from the Materials Project and Pymatgen databases.[1,2] The metal surface structure information descriptors, which include the coordination number of the single-atom metal in the topmost layer and the total coordination number, were obtained by automatically cutting and analyzing surfaces using Pymatgen.[2] Both the BrunnerNN_real and MinimumDistanceNN methods were used to analyze the coordination number. Moreover, based on the position of the coordinated atoms on the metal surface, the coordination number within the top 0.2 radius (CN-B3+1-top02), top 0.5 radius (CN-B3+1-top), top 1 radius (CN-B3+1-top05), and the total coordination number (CN-B3+1) were obtained. The newly created descriptors are a combination of the properties of single-atom metal and its host metal. After eliminating descriptors with missing values, we obtained a total of 85 feature descriptors, all of which were automatically generated, requiring no additional theoretical calculations.

The performance of feature sets was evaluated by the Pearson correlation coefficient (p), which is determined by

$$p=\frac{\Sigma_{\mathrm{i}}(f_i-\bar{f})(F_i-\bar{F})}{\sqrt{\Sigma_{\mathrm{i}}(f_i-\bar{f})^2}\sqrt{\Sigma_{\mathrm{i}}(F_i-\bar{F})^2}} \tag{3}$$

in which f_i and F_i are the compared features. The value of p is in the range of −1.0 to 1.0, and a higher absolute value of p presents a stronger correlation.

The Pearson correlation coefficients of these 85 descriptors were shown in Fig. 1b, where some descriptors exhibited high correlation, resulting in redundant information. Therefore, the mutual information regression (MIC) were performed to screen the highly correlated features by the rank of feature importance. Then, the Recursive Feature Elimination with Cross-Validation (RFECV) method[3] was employed to

further refine the feature selection process. The RFECV method could systematically eliminate less important features, thereby streamlining the model. The incorporation of cross-validation provided a robust mechanism for assessing feature relevance, reducing overfitting and improving model generalizability. Both methods are implemented through the Scikit-Learn package.[4] After screening, the Pearson correlation coefficient between all selected descriptors is less than 0.8.

Mutual information is defined as:

$$I(X;Y)= D_{KL}||P_X \otimes P_Y \tag{4}$$

where (X, Y) is a pair of random variables with values over the space (X x Y), $P_{(X, Y)}$ is their joint distribution, P_X and P_Y are the marginal distributions, D_{KL} is the Kullback-Leibler divergence.

Model Selection

Different machine learning (ML) methods show different prediction performances even if the same feature set and training data are used. In this study, we employed 9 different ML classification algorithms, i.e., multilayer perceptron classifier (MLPC)[5], gradient boosting classifier (GBC), random forest classifier (RFC), extra tree classifier (ETC), decision tree classifier (DTC), kneighbors classifier (KNC), linear support vector classifier (LSVC), and ridge classifier (RidgeC), and 15 different ML regression algorithms, i.e., multilayer perceptron regression (MLP), extreme gradient boosting regression (XGB)[6], gradient boosting regression (GBDT), random forest regression (RF), extra tree regression (ETR), adaptive boost regression (ADAB), linear regression (LR), ridge regression (RIDGE), lasso regression (LAS), bayesian ridge regression (BAY), bayesian ARD regression (ARD), Gaussian process regression (GPR), linear support vector regression (LSVR), support vector regression (SVR), and k-neighbor regression (KNN). Aside from XGB, all other methods are

implemented through the Scikit-Learn package.

Firstly, hyperparameter optimization for these models was performed via a grid search method based on the training set from random sampling. This involved randomly sampling hyperparameter combinations within a defined search space for each algorithm. The purpose was to find the optimal hyperparameters that yield the best performance on the test data. This process helped to explore a wide range of hyperparameter settings and find out the ones that maximize the model's predictive capabilities.

After the optimal hyperparameters were determined, we evaluated the performance of the trained ML models, including predictive accuracy and generalization capabilities. Each training model was based on a random subset of 80% of the whole data set, known as a training set, and the model would be used for the prediction of the unused data, known as a test set. As the accuracy and difficulty of the prediction are highly dependent on the training and test splits, the training procedure was executed 1000 times for each ML regression algorithm in order to eliminate the impact of randomness. To evaluate the performance of each ML regression model, the coefficient of determination (r^2), mean absolute error (MAE) and root mean square error (RMSE) were calculated to estimate the prediction errors. Moreover, the mean, minimum and maximum values of these three indexes were used as the criterion. The model we wanted is that which performs well and possesses small fluctuations simultaneously under a large number base.

The r^2, RMSE, and MAE are defined as:

$$r^2 = 1 - \frac{\sum_i (Y_i - y_i)^2}{\sum_i (Y_i - \bar{Y})^2} \tag{5}$$

$$RMSE=\sqrt{\frac{1}{N}\sum_{i=1}^{N}(Y_i\text{-}y_i)^2} \quad (6)$$

$$MAE=\frac{1}{N}\sum_{i=1}^{N}|Y_i\text{-}y_i| \quad (7)$$

where Y_i indicates the value of the DFT computations, y_i indicates the result predicted by the ML models, and $\overline{Y}$ indicates the average value of the DFT data. Generally, an ideal model should present an r^2 value close to 1, and a small RMSE and MAE close to 0.

For classification, it should be noted that there might exist multiple reaction pathways on a single SAA surface, thus a multi-label classification method allowing for multiple reaction pathways on one SAA was adopted. Besides, to prevent omission, the recall rate was set as the sole evaluation criterion for the ML model, disregarding its precision. Furthermore, the top two models, ETC and KNC, which have the highest recall scores, were used for classification prediction. The labels of SAAs surfaces predicted by the two methods were summed to further avoid the possibility of omission. The classifical results of the 9 model on the test set were listed in Supplementary Table 4.

The recall score is defined as:

$$Recall=\frac{TP}{TP+TN} \quad (8)$$

where TP is true positive, TN is true negative.

Results

Through DFT computations, we obtained 623 energy barrier data for C-H dissociation. Initially, ML training were directly conducted on these data. After feature engineering, 18 descriptors were selected. Then, a hyperparameter search was

conducted for various regression algorithms. The hyperparameter search space and the best hyperparameters could be found in Supplementary Table 3. Subsequently, 1000 different splits of the training set and test set were estimated to test the trained ML model. The results of the 1000 tests could be found in Supplementary Fig. 8, where the GBDT model performed best. In 1000 trials, the average r^2, RMSE, and MAE of GBDT are 0.841, 0.197 eV, and 0.136 eV, respectively.

Upon analysis, the inadequate performance of ML was due to the varied dissociation methods of CH on different metal surfaces. As illustrated in Supplementary Fig. 3, the hydrogen in the transition state is situated at the top site of the single-atom metal on host metals such as Ni, Cu, Pd, etc. On host metals like Sc, Ti, Zn, etc., the hydrogen in the transition state is located at the bridge site of the host metal and the single-atom metal. In certain SAA surfaces, the hydrogen in the transition state is positioned on the bridge or hollow site of the two host metals. Different hydrogen adsorption sites correspond to different reaction mechanisms, which should, in turn, correspond to different descriptors and relationships. Therefore, we first manually categorized these structures, then used ML methods to classify the prediction set, and conducted regression predictions separately for the C-H dissociation energy barriers of different reaction mechanisms.

A multi-label classification method allowing for multiple reaction pathways on one SAA were adopted. The scores of different classification methods and their hyperparameter settings were shown in Supplementary Table 2. The ETC and KNC models were both used for classification prediction and then the predictions of them are summed. This approach can further avoid omissions. After classification, 353 training set data and 8638 prediction set data for the top site reaction pathway; 182 training set data and 3988 prediction set data for the bridge site reaction pathway, and

88 training set data and 982 prediction set data for other site reaction pathways were obtained. Following this, ML regression training were carried out for the C-H dissociation energy barriers of the top and bridge site reaction pathways, respectively.

For the pathway of H at the top site, first, we evaluated the performance of various ML models using all 84 features as descriptors without feature selection. Hyperparameters for all models were optimized using the Random Search method. The results are shown in Supplementary Fig. 9. In 300 different splits of training and test sets, the MLP model performed the best. Its r^2, RMSE, and MAE are 0.905, 0.145 eV, and 0.101 eV, respectively. Then, we conducted feature selection for the descriptors. After feature selection, 24 features were obtained. Then, a random search for hyperparameters for the 15 ML regression algorithms were conducted. The best hyperparameters for each algorithm were shown in Supplementary Table 3. Subsequently, the trained models with 1000 different splits of training and test sets were tested. The scores of each algorithm in the training set were nearly perfect. The violin plots for the distribution of RMSE, MAE, and r^2 for each ML algorithm in the 1000 trials in the test set were shown in Supplementary Fig. 9. Among them, MLP and GBDT demonstrated excellent performance. The average values of r^2, RMSE, and MAE for MLP in the 1000 trials on the test set were 0.921, 0.130 eV, and 0.094 eV, respectively. The average values of r^2, RMSE, and MAE for GBDT in the 1000 trials on the test set were 0.913, 0.136, and 0.100 eV, respectively. However, the MLP model performed poorly in the other two datasets, indicating limited generalization capability. Therefore, we chose the GBDT model, which exhibited superior performance across all three datasets, to predict the C-H dissociation energy barrier on the 8,638 SAA surfaces. The prediction results were shown in Fig.s 1 and S12.

The ML training process for the pathway of H at the bridge site follows the same

steps as the pathway at the top site. After feature engineering, 26 features were screened. Then, a hyperparameter search was conducted, and the best hyperparameters were shown in Supplementary Table 3. The trained models were trialed with 1000 different training and test set splits. As shown in Supplementary Fig. 10, GBDT showed the best performance with an average r^2, RMSE, and MAE of 0.762, 0.133 eV, and 0.095 eV, respectively, in the 1000 trials. However, due to the limited number of data points in the training set, the r^2 of the GBDT model was too low and the RMSE fluctuated significantly, making the model performance unreliable. Therefore, we did not proceed with a prediction for the pathway of H at the bridge site.

1.6 Preparation of the M/Fe (M =Re, Ir, Rh, Pt, Ru)

Fe balls, weighing 333 g with diameters of 8–10 mm, were procured from Aladdin. Before loading metals, the Fe balls were sonicated in N,N-Dimethylformamide (DMF) for 30 min and washed with deionized water. After drying, they were immersed in a solution of perrhenic acid (0.0127 M) for 10 h. Subsequently, filtration, air-drying for 30 min, calcination at 500 ℃, and reduction at 500 ℃ were performed under an atmosphere of 20 mL/min 10% H_2/Ar. For the loading of Ir/Fe, Rh/Fe, Pt/Fe and Ru/Fe, chloroiridic acid (0.0127 M), rhodium acetylacetonate (0.0127 M), chloroplatinic acid (0.0127 M) and ruthenium acetylacetonate (0.0127 M) were used as precursors with similar loading procedures.

1.7 Rolling ball milling experiment

200 g Ir/Ni was sealed in the reactor, and the temperature was controlled by a K-type thermocouple inserted into the reactor. The feed gas is pure CH_4 with a flow rate of 50 mL/min. The reaction is carried out at atmospheric pressure. The products

were analyzed by on-line gas chromatography (ThermoFisher Trace1300).=

1.8 Conversion and Production rates calculation

$$CH_4 conversion = \frac{CH_4\ inlet - CH_4\ outlet}{CH_4\ inlet} \times 100\% \quad (9)$$

where CH_4 inlet and CH_4 outlet present moles of CH_4 at the inlets and outlets, respectively.

$$H_2\ production = \frac{CH_4\ conversion \times CH_4\ flow \times 2}{m_{catalyst}} \quad (10)$$

where CH_4 flow represents the flow rate of CH_4, $m_{catalyst}$ is the mass of active metal atoms on the catalyst.

2. Supplementary Table

Supplementary Table 1. 85 descriptions and their types and sources.

Number	Descriptor	Type	Source
0	host_surface_energy	1	Materials project
1	host_surface_work_function		Materials project
2	host_surface_efermi		Materials project
3	CN-M3+1	2	Pymatgen.MinimumDistanceNN
4	CN-B3+1		Pymatgen.BrunnerNN_real
5	CN-B3+1-top		Pymatgen.BrunnerNN_real
6	CN-B3+1-top05		Pymatgen.BrunnerNN_real
7	CN-B3+1-top02		Pymatgen.BrunnerNN_real
8	host_symmetry_number	1	Materials project
9	host_volume		Materials project
10	host_density		Materials project
11	host_efermi		Materials project
12	host_shape_factor		Materials project
13	host_total_magnetization		Materials project
14	host_weighted_surface_energy		Materials project

15	host_weighted_work_function	Materials project
16	host_out_e_number	Materials project
17	host_d_e_number	Materials project
18	host_period	Materials project
19	host_HOMO	Materials project
20	host_LUMO	Materials project
21	host_cohesive_energy	Materials project
22	host_d-band_center	Materials project
23	host_number	Pymatgen
24	host_atomic_radius_calculated	Pymatgen
25	host_van_der_waals_radius	Pymatgen
26	host_mendeleev_no	Pymatgen
27	host_electrical_resistivity	Pymatgen
28	host_molar_volume	Pymatgen
29	host_thermal_conductivity	Pymatgen
30	host_boiling_point	Pymatgen
31	host_melting_point	Pymatgen
32	host_liquid_range	Pymatgen
33	host_ionization_energies_1	Pymatgen
34	host_ionization_energies_2	Pymatgen
35	host_ionization_energies_3	Pymatgen
36	host_electronegativity	Pymatgen
37	host_atomic_mass	Pymatgen
38	host_atomic_radius	Pymatgen
39	host_anerage_ionic_radius	Pymatgen
40	host_electron_affinity	Pymatgen
41	doped_volume	Materials project
42	doped_density	Materials project
43	doped_efermi	Materials project
44	doped_shape_factor	Materials project

45	doped_total_magnetization		Materials project
46	doped_weighted_surface_energy		Materials project
47	doped_weighted_work_function		Materials project
48	doped_out_e_number		Materials project
49	doped_d_e_number		Materials project
50	doped_period		Materials project
51	doped_HOMO		Materials project
52	doped_LUMO		Materials project
53	doped_cohesive_energy		Materials project
54	doped_d-band_center		Materials project
55	doped_number		Pymatgen
56	doped_atomic_radius_calculated		Pymatgen
57	doped_van_der_waals_radius		Pymatgen
58	doped_mendeleev_no		Pymatgen
59	doped_electrical_resistivity		Pymatgen
60	doped_molar_volume		Pymatgen
61	doped_thermal_conductivity		Pymatgen
62	doped_boiling_point		Pymatgen
63	doped_melting_point		Pymatgen
64	doped_liquid_range		Pymatgen
65	doped_ionization_energies_1		Pymatgen
66	doped_ionization_energies_2		Pymatgen
67	doped_ionization_energies_3		Pymatgen
68	doped_electronegativity		Pymatgen
69	doped_atomic_mass		Pymatgen
70	doped_atomic_radius		Pymatgen
71	doped_anerage_ionic_radius		Pymatgen
72	doped_electron_affinity		Pymatgen
73	com_top_d-band	3	(num(54)+num(22)*num(7))/(num(7)+1)
74	com_all_d-band		(num(54)+num(22)*num(3))/(num(3)+1)

75	com_all_electronegativity	(num(68)+num(36)*num(3))/(num(3)+1)
76	com_top_electronegativity	(num(68)+num(36)*num(7))/(num(7)+1)
77	com_cha_electronegativity	num(68)-num(36)*num(3)
78	com_cha2_electronegativity	num(68)-(num(68)-num(22))*num(3))
79	com_all_out_e_number	num(48)+num(16)*num(3)
80	com_top_out_e_number	num(48)+num(16)*num(7)
81	com_all_d_e_number	num(49)+num(17)*num(3)
82	com_top_d_e_number	num(49)+num(17)*num(7)
83	rd-rh	num(70)-num(38)
84	rd/rh	num(70)/num(38)

Supplementary Table 2. Hyperparameter search space of ML classification model and hyperparameter corresponding to the optimal model.

Model	Hyperparameter range	Best hyperparameter
MLPC	{"hidden_layer_sizes":[400,600,800,1000,(1000,200),(1000,500),(1000,1000,500)], "activation":["relu","logistic","tanh"], "learning_rate_init":loguniform.rvs(0.001,0.1,size=5), "alpha":loguniform.rvs(0.0001,0.1,size=5), "solver":['lbfgs','sgd','adam']}	{'solver':'sgd', 'learning_rate_init':0.0012550180809854483, 'hidden_layer_sizes':(1000,200), 'alpha':0.0010305247237014014, 'activation': 'tanh'}
GBC	{"estimator__subsample":np.linspace(0.5,1,6), "estimator__loss": ['log_loss','exponential'], "estimator__min_samples_split":[2,3,4,5], "estimator__learning_rate":loguniform.rvs(0.001,0.2,size=5), "estimator__n_estimators":range(100,1100,100), "estimator__max_depth":range(1,11,1),}	{'estimator__subsample':0.7, 'estimator__n_estimators':500, 'estimator__min_samples_split':3, 'estimator__max_depth': 1, 'estimator__loss':'log_loss', 'estimator__learning_rate':0.10335330474527947}
RF	{"n_estimators":range(100,1100,100),	{'n_estimators':800,

C	"max_depth":range(1,11,1), "bootstrap":[True,False], "max_features":['sqrt','log2',30,45,60,80,100], "min_samples_split":range(2,10,1)}	'min_samples_split':4, 'max_features':'sqrt', 'max_depth': 8, 'bootstrap': True}
ETC	{"n_estimators":range(100,1100,100), "max_depth":range(1,11,1), "bootstrap":[True,False], "max_features":['sqrt','log2',30,45,60,80,100], "min_samples_split":range(2,10,1)}	{'n_estimators':700, 'min_samples_split':5, 'max_features':'log2', 'max_depth': 10, 'bootstrap': False}
DTC	{"criterion":['gini','entropy','log_loss'], "splitter":['best','random'], "max_depth":range(1,11,1), "max_features":['sqrt','log2',30,45,60,80,100], "min_samples_split":range(2,10,1)}	{'splitter':'random', 'min_samples_split':6, 'max_features':80, 'max_depth':5, 'criterion': 'entropy'}
KNC	{"n_neighbors":range(2,21,2), "weights":["uniform",'distance'], "leaf_size":range(20,51,10)}	{'weights':'distance', 'n_neighbors':16, 'leaf_size': 40}

Supplementary Table 3. Hyperparameter search space of ML regression model and hyperparameter corresponding to the optimal model.

Model	Hyperparameter range	Best hyperparameter for all-path	Best hyperparameter for CH on the top site	Best hyperparameter for CH on the bridge site
MLP	{"hidden_layer_sizes":[400,600,800,1000, (1000,200),(1000,500),(1000,1000,500)], "activation":["relu","logistic","tanh"], ”learning_rate_init":logu	{'solver':'adam', 'learning_rate_init':0.01027026196551456, 'hidden_layer_sizes': (1000,500), 'alpha':0.001556190	{'solver':'lbfgs', 'learning_rate_init':0.036039868059876176, 'hidden_layer_sizes': (1000, 200), 'alpha':0.072990857	{'solver':'lbfgs', 'learning_rate_init':0.006076474667372711, 'hidden_layer_sizes': 600, 'alpha':0.002199815

	niform.rvs(0.001,0.1,size=5), "alpha":loguniform.rvs(0.0001,0.1,size=5), "solver":['lbfgs','sgd','adam']}	5795767584, 'activation': 'relu'}	22491738, 'activation':'relu'}	694585654, 'activation': 'relu'}
XGB	"learning_rate":loguniform.rvs(0.001,0.5,size=5), "n_estimators":range(100,1100,100), "max_depth":range(2,12,2)}	{'n_estimators': 600, 'max_depth':6, 'learning_rate': 0.026093585831582042}	{'n_estimators': 500, 'max_depth':4, 'learning_rate': 0.04302624321212522}	{'n_estimators': 600, 'max_depth':2, 'learning_rate': 0.06302809944884195}
GBDT	{"subsample":np.linspace(0.5,1,6), "loss":["squared_error", "absolute_error", "huber"], "min_samples_split":[2,3,4,5], "learning_rate":loguniform.rvs(0.001,0.2,size=5), "n_estimators":range(100,1100,100), "max_depth":range(1,11,1),}	{'subsample':0.7, 'n_estimators':500, 'min_samples_split': 4, 'max_depth':9, 'loss':'absolute_error', 'learning_rate': 0.0430431714851817}	{'subsample':0.8, 'n_estimators':500, 'min_samples_split': 3, 'max_depth': 3, 'loss': 'squared_error', 'learning_rate': 0.09239156092984442}	{'subsample': 0.5, 'n_estimators': 900, 'min_samples_split': 3, 'max_depth': 4, 'loss': 'squared_error', 'learning_rate': 0.020030214379475116}
RF	{"n_estimators":range(100,1100,100), "max_depth":range(1,11,1), "bootstrap":[True,False],	{'n_estimators': 700, 'min_samples_split': 2, 'max_features': 45, 'max_depth': 10,	{'n_estimators': 400, 'min_samples_split': 6, 'max_features': 30, 'max_depth': 10,	{'n_estimators': 300, 'min_samples_split': 3, 'max_features': 1.0, 'max_depth': 9,

	"max_features":[1.0,30,45,60,80,100], "min_samples_split":range(2,10,1)}	'bootstrap': True}	'bootstrap': True}	'bootstrap': True}
ETR	{"n_estimators":range(100,1100,100), "max_depth":range(1,11,1), "bootstrap":[True,False], "max_features":[1.0,30,45,60,80,100], "min_samples_split":range(2,10,1)}	{'n_estimators': 600, 'min_samples_split': 2, 'max_features': 45, 'max_depth': 10, 'bootstrap': False}	{'n_estimators': 400, 'min_samples_split': 8, 'max_features': 60, 'max_depth': 10, 'bootstrap': False}	{'n_estimators': 900, 'min_samples_split': 4, 'max_features': 100, 'max_depth': 9, 'bootstrap': False}
ADAB	{"n_estimators":range(100,1100,100), "learning_rate":loguniform.rvs(0.001,0.1,size=5), "loss":['linear','square','exponential']}	{'n_estimators': 800, 'loss': 'square', 'learning_rate': 0.08516725629944769}	{'n_estimators': 700, 'loss': 'square', 'learning_rate': 0.040633361534761825}	{'n_estimators': 900, 'loss': 'linear', 'learning_rate': 0.07627389453447658}
BAY	{"alpha_1":[1e-04,1e-06,1e-08], "alpha_2":[1e-04,1e-06,1e-08], "lambda_1":[1e-04,1e-06,1e-08], "lambda_2":[1e-04,1e-06,1e-08]}	{'lambda_2': 0.0001, 'lambda_1': 1e-08, 'alpha_2': 1e-08, 'alpha_1': 1e-08}	{'lambda_2': 0.0001, 'lambda_1': 0.0001, 'alpha_2': 0.0001, 'alpha_1': 1e-06}	{'lambda_2': 0.0001, 'lambda_1': 1e-06, 'alpha_2': 1e-08, 'alpha_1': 0.0001}
ARD	{"alpha_1":[1e-04,1e-06,1e-08], "alpha_2":[1e-04,1e-06,1	{'lambda_2': 0.0001, 'lambda_1': 1e-08, 'alpha_2': 1e-08,	{'lambda_2': 0.0001, 'lambda_1': 1e-08, 'alpha_2': 1e-06,	{'lambda_2': 0.0001, 'lambda_1': 1e-08, 'alpha_2': 1e-08,

	e-08], "lambda_1":[1e-04,1e-06, 1e-08], "lambda_2":[1e-04,1e-06, 1e-08]}	'alpha_1': 0.0001}	'alpha_1': 0.0001}	'alpha_1': 0.0001}
GPR	{"kernel":[ConstantKernel(1.0, (1e-5, 1e5)) * RBF(1.0, (1e-5, 1e5)) + WhiteKernel(1.0, (1e-5, 1e5)), ConstantKernel(0.5, (1e-5, 1e5)) * RBF(1.0, (1e-5, 1e5)) + WhiteKernel(1.0, (1e-5, 1e5)), ConstantKernel(0.5, (1e-5, 1e5)) * RBF(0.5, (1e-5, 1e5)) + WhiteKernel(0.5, (1e-5, 1e5)), ConstantKernel(0.5, (1e-5, 1e5)) * RBF(0.5, (1e-5, 1e5)), ConstantKernel(0.5, (1e-5, 1e5)) * RBF(0.1, (1e-5, 1e5)), ConstantKernel(0.1, (1e-5, 1e5)) * RBF(0.1, (1e-5, 1e5)) + WhiteKernel(0.1, (1e-5,	{'kernel': 1**2 * RBF(length_scale=1) + WhiteKernel(noise_level=1), 'alpha': 2.0536746924733707e-08}	{'kernel': 1**2 * RBF(length_scale=1) + WhiteKernel(noise_level=1), 'alpha': 4.344309524149596e-07}	{'kernel': 0.707**2 * RBF(length_scale=1) + WhiteKernel(noise_level=1), 'alpha': 3.5739805888619224e-09}

	1e5)), None], "alpha" : loguniform.rvs(1e-10,1e-5,size=5)}			
SVR	{"kernel":['rbf','poly','linear'], "gamma":loguniform.rvs(1e-6,1e-2,size=5), "C":loguniform.rvs(0.1,1e5,size=8), "shrinking":[True,False]}	{'shrinking': True, 'kernel': 'rbf', 'gamma': 0.00663728377701768, 'C': 244.38972414635543}	{'shrinking': True, 'kernel': 'rbf', 'gamma': 0.0015608721432150104, 'C': 1065.8347822023532}	{'shrinking': True, 'kernel': 'rbf', 'gamma': 4.35519499451895e-05, 'C': 2273.468871250735}
KNN	{"n_neighbors":range(2,21,2), "weights":["uniform",'distance'], "leaf_size":range(20,51,10)}	{'weights': 'distance', 'n_neighbors': 4, 'leaf_size': 50}	{'weights': 'distance', 'n_neighbors': 6, 'leaf_size': 20}	{'weights': 'distance', 'n_neighbors': 8, 'leaf_size': 50}

Supplementary Table 4. Classification results of 9 ML models on test set

Model	Recall	Precision
MLPC	0.90	0.91
GBC	0.90	0.90
RFC	0.90	0.90
ETC	0.92	0.90
DTC	0.86	0.85

RidgeC	0.87	0.87
LSVC	0.89	0.88
KNC	0.91	0.89
ETC+KNC	0.93	0.84

Supplementary Table 5. The CH_4 cracking performance on various SAAs.[a]

Catalyst	H_2 yield/gH_2 gCat^{-1} h^{-1}
Ru/Fe	0.091
Re/Fe	0.028
Ir/Fe	0.127
Rh/Fe	0.101
Pt/Fe	0.068
Ru/Ni	2.09
Re/Ni	10.4
Ir/Ni	13.3

a Reaction conditions: 0.1 MPa; 450 ℃; 50 mL/min CH_4; 500 rpm motor speed.

Supplementary Table 6. Comparison of hydrogen production performance from CH_4 cracking

Catalyst	T/°C	H_2 yield/g_{H2} g_{Cat}^{-1} h^{-1}	time/h	ref
Ni–Fe/Al_2O_3	650	0.892	210	7
Ni–Pd/Al_2O_3	750	0.579	10	8
Fe-Co/Al_2O_3	700	0.38	3	9

Ni-Co/SBA-15	700	0.171	5	10
Ni-Bi	1065	--	170	11
Ni–Cu/CNT	700	4.4	30	12
Ni–Cu/SiO_2	650	5.333	30	13
Ni–Cu/Al_2O_3	700	16	2.5	14
NiMo-Bi	450	0.001	--	15
NiMo-Bi	800	0.022	120	15
Ni-Cu-Co	700	0.809	6	16
Re/Ni	450	10.4	240	This work
Ir/Ni	450	13.3	--	This work

Supplementary Table 7. EXAFS fitting parameters at the Re L-edge for various samples（S_0^2=0.7）

Sample	Shell	CN^a	R(Å)^b	σ^2(Å^2)^c	ΔE_0(eV)^d	*R* factor
Re foil	Re-Re	12*	2.75±0.005	0.004±0.0006	6.60±1.00	0.007
Sample Re	Re-Ni	7.4±0.6	2.52±0.003	0.003±0.0004	10.83±0.72	0.004

$^a CN$, coordination number; $^b R$, the distance between absorber and backscatter atoms; $^c\sigma^2$, Debye-Waller factor to account for both thermal and structural disorders; $^d\Delta E_0$, inner potential correction; *R* factor indicates the goodness of the fit. $S0^2$ was fixed to 0.7, according to the experimental extended X-ray absorption fine structure (EXAFS) fit of Re foil by fixing CN as the known crystallographic value.

Supplementary Table 8. EXAFS fitting parameters at the Ir L-edge for various samples（S_0^2=0.8）

Sample	Shell	CN^a	R(Å)^b	σ^2(Å^2)^c	ΔE_0(eV)^d	*R* factor
Ir foil	Ir-Ir	12*	2.74±0.005	0.003±0.0005	8.99±0.51	0.004
Sample Ir-1	Ir-Ni	9.32±1.1	2.47±0.007	0.008±0.0009	5.41±1.24	0.009

$^a CN$, coordination number; $^b R$, the distance between absorber and backscatter atoms; $^c\sigma^2$, Debye-Waller factor to account for both thermal and structural disorders; $^d\Delta E_0$, inner potential correction; *R* factor indicates the goodness of the fit. $S0^2$ was fixed to 0.8, according to the experimental EXAFS fit of Ir foil by fixing CN as the known crystallographic value.

3. Supplementary Figures

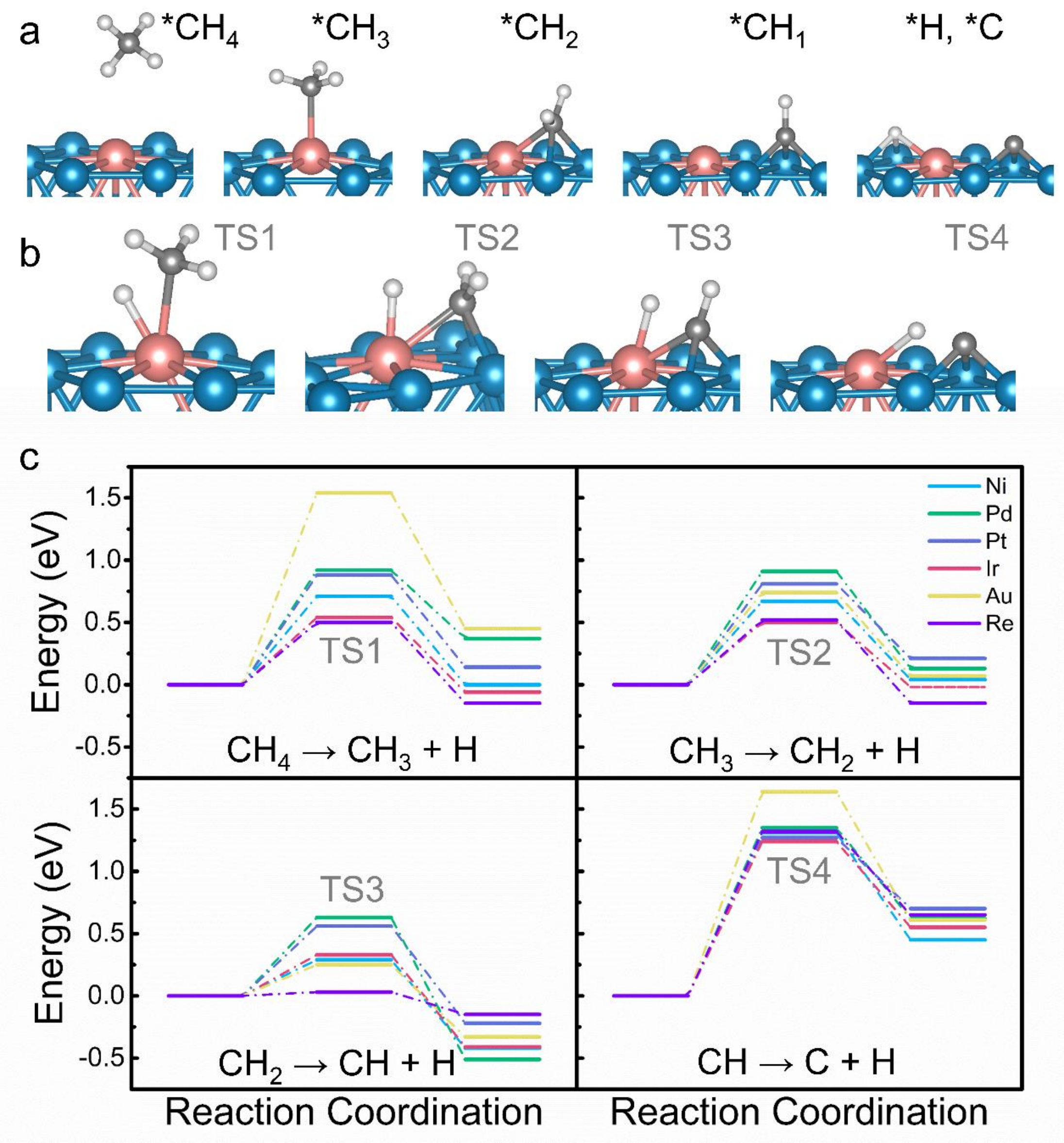

Supplementary Fig. 1 Energy barriers and corresponding intermediate structure diagrams for the four-step dehydrogenation of CH_4 on different atoms doped Ni (111) surface.

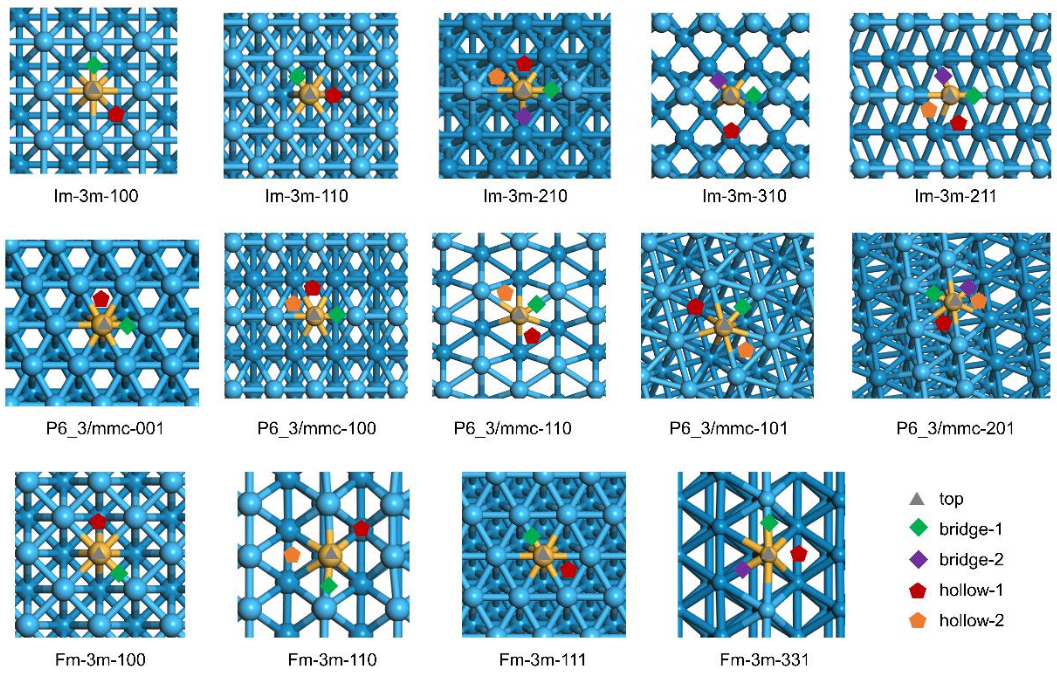

Supplementary Fig. 2 Illustration of adsorption sites on different surfaces across various space groups.

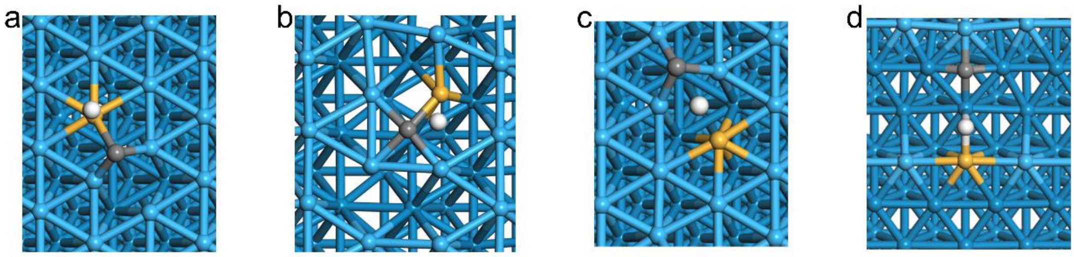

Supplementary Fig. 3 Structural diagrams of the transition state of H at different positions; (a) top position, (b) bridge position, and (c & d) hollow positions.

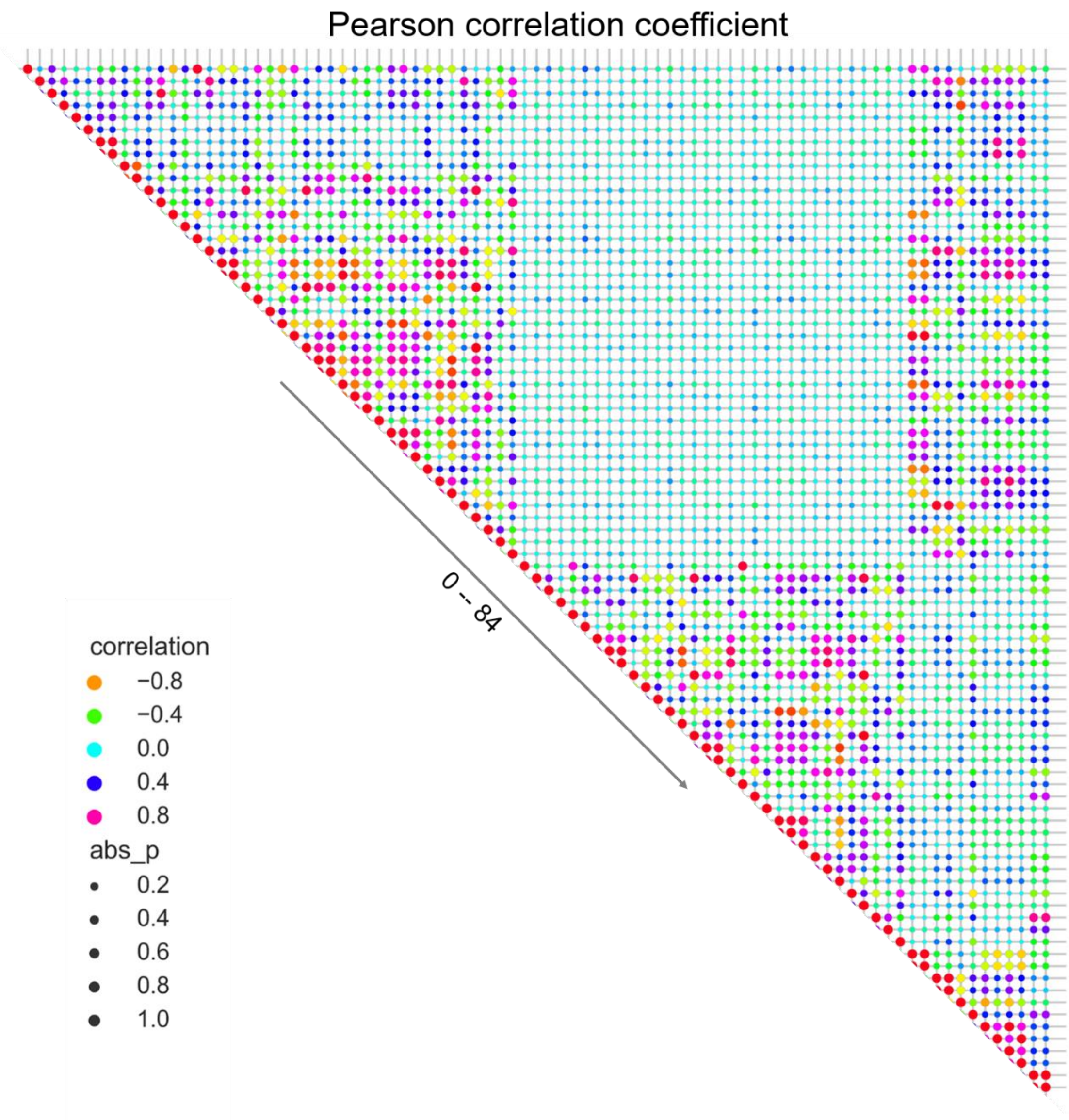

Supplementary Fig. 4 Pearson correlation coefficient plot for the 84 descriptors.

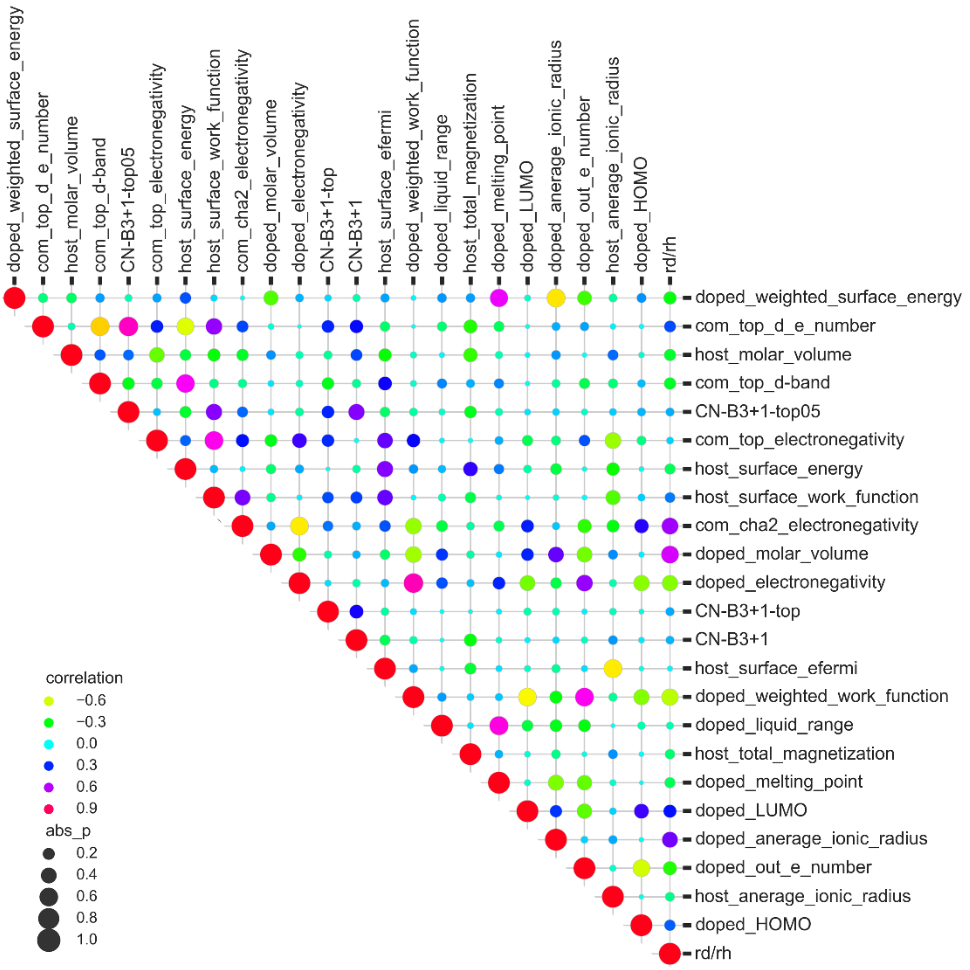

Supplementary Fig. 5 Pearson correlation coefficient plot for the 24 descriptors of H at the top position.

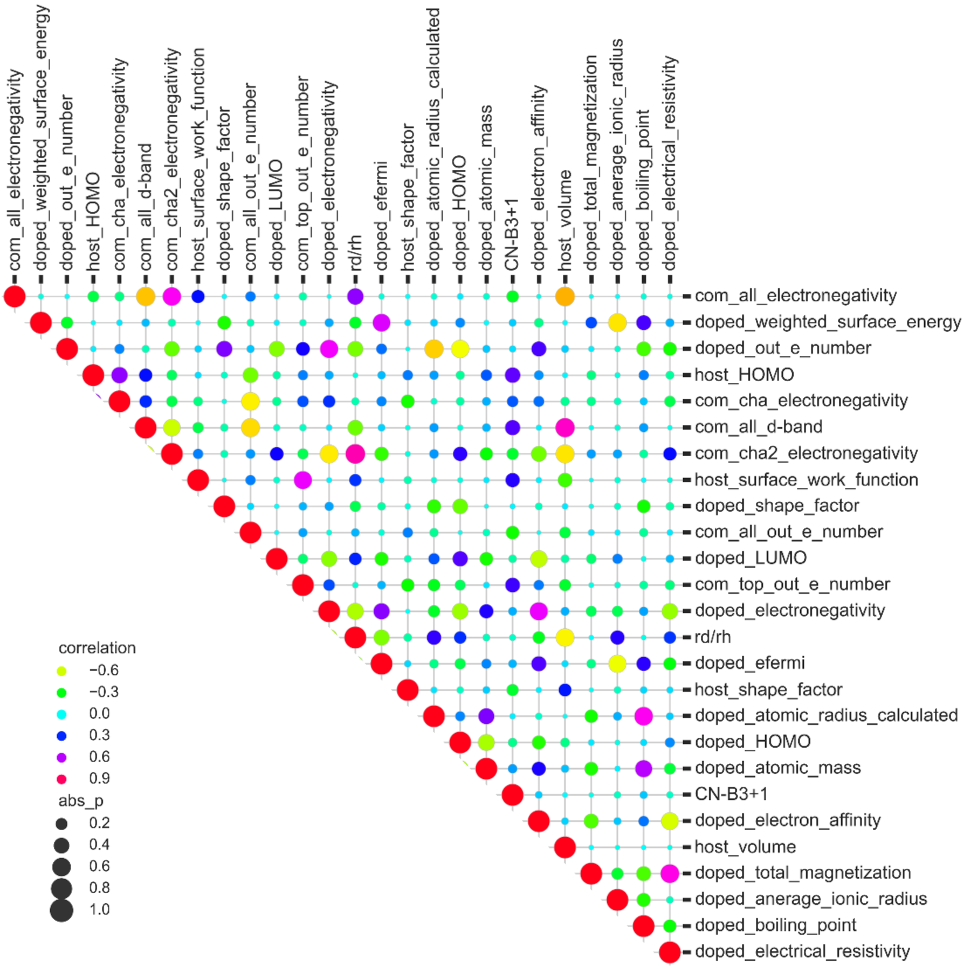

Supplementary Fig. 6 Pearson correlation coefficient plot for the 26 descriptors of H at the bridge position.

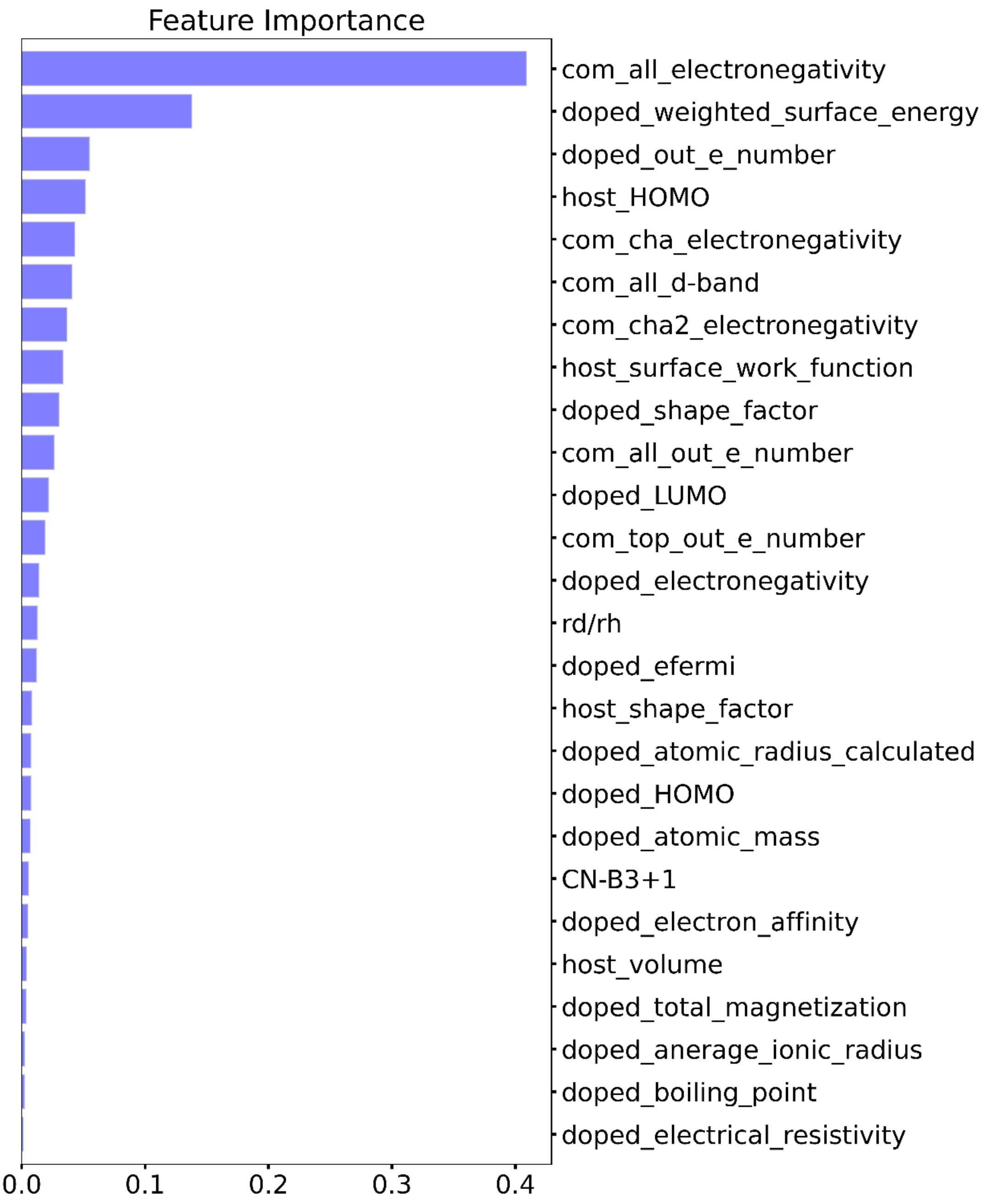

Supplementary Fig. 7 Feature importance ranking for the 26 descriptors of H at the bridge position.

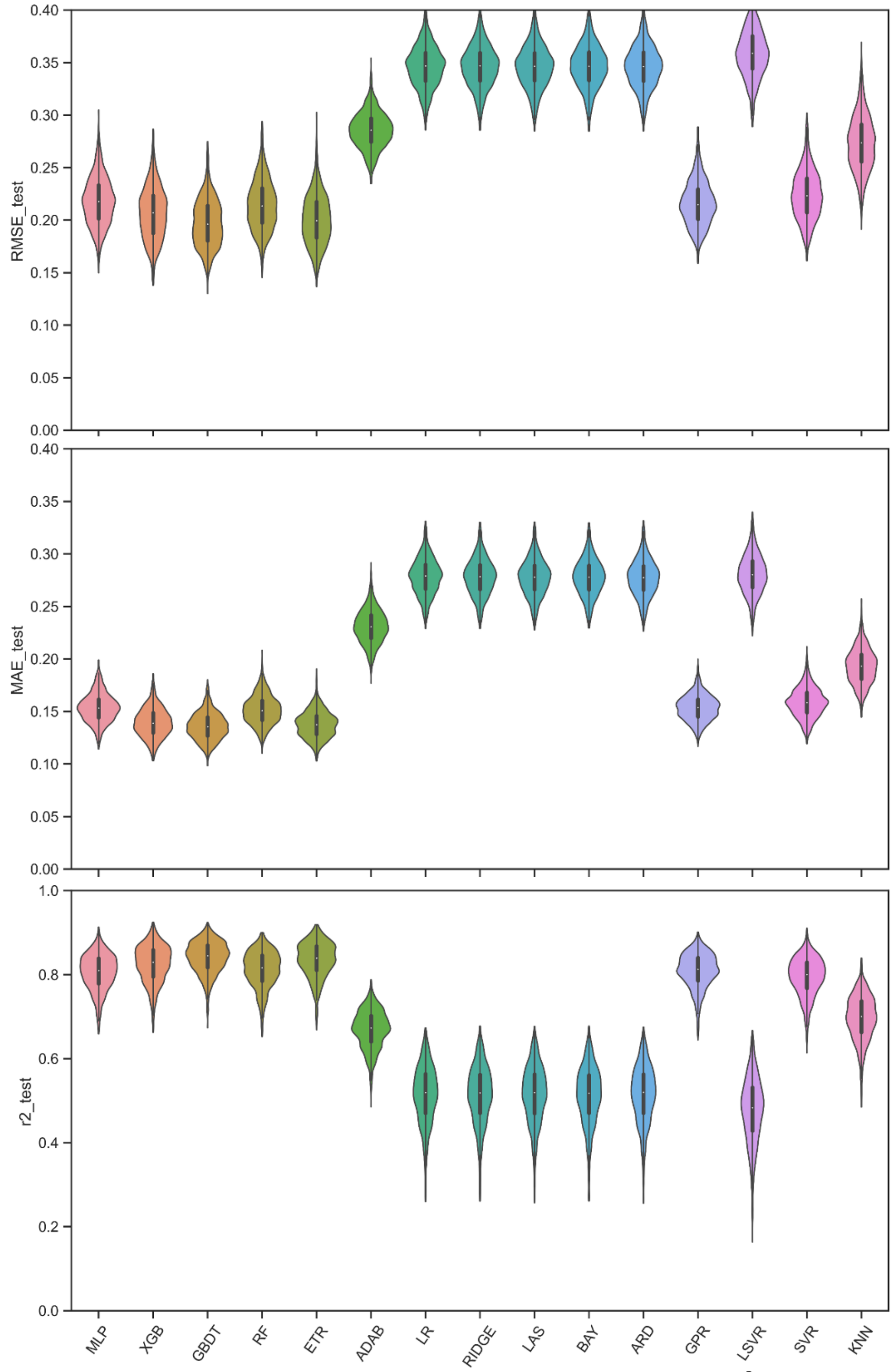

Supplementary Fig. 8 Violin plots for the distribution of RMSE, MAE, and r^2 for each ML algorithm in the 1000 trials in the test set without classification dataset.

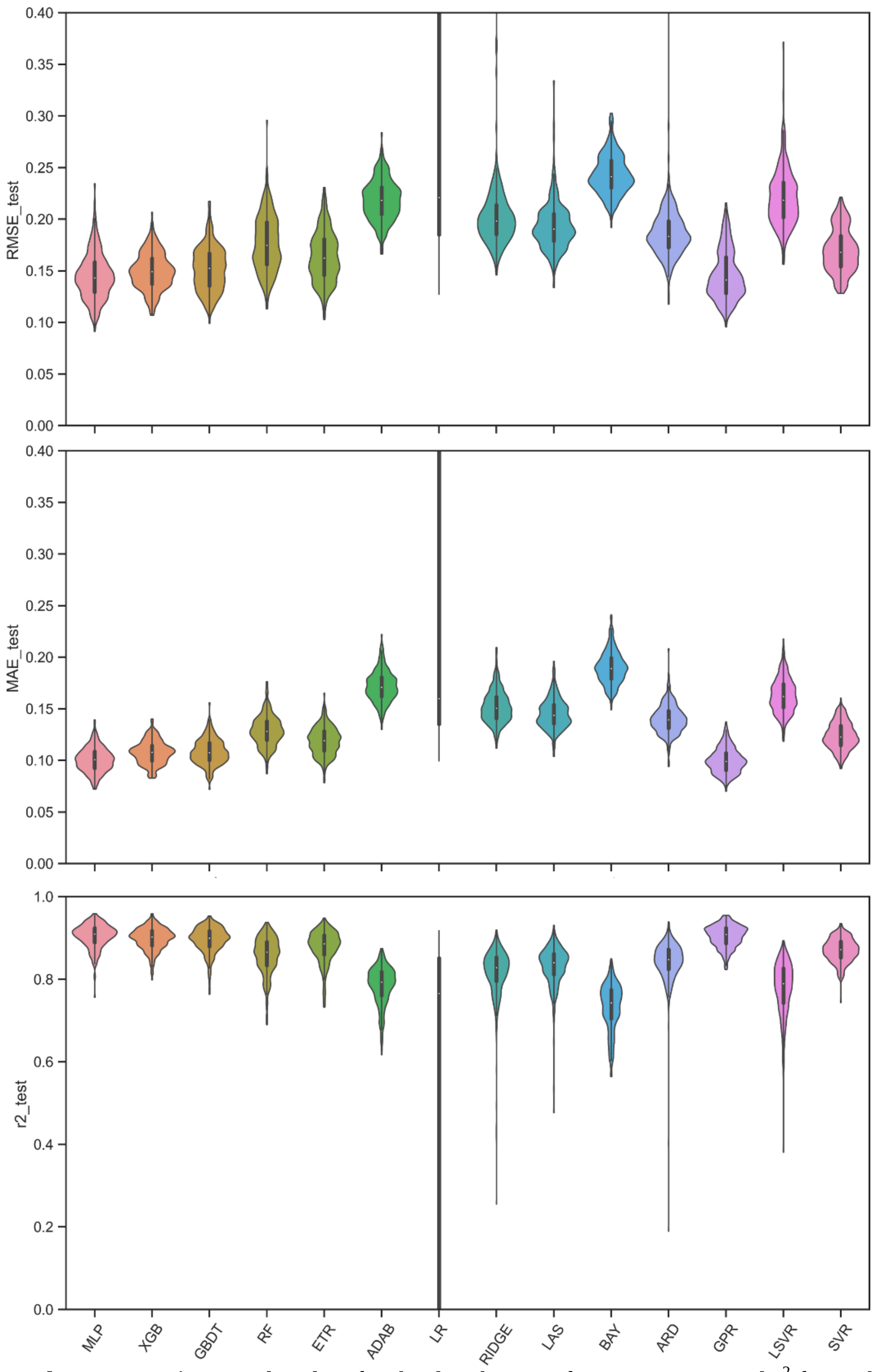

Supplementary Fig. 9 Violin plots for the distribution of RMSE, MAE, and r^2 for each ML algorithm in the 300 trials in the test set of H at top sites dataset without feature selection.

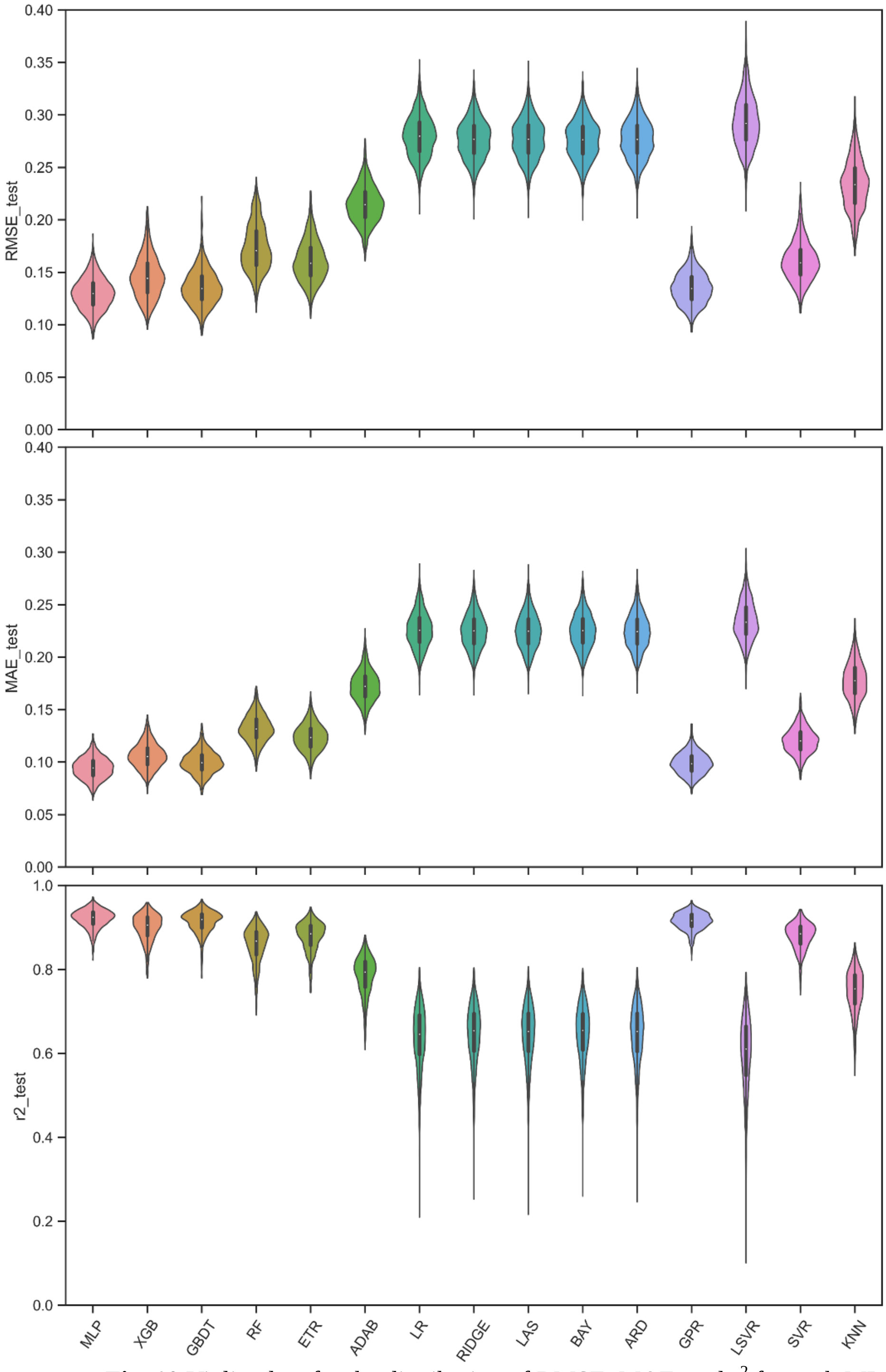

Supplementary Fig. 10 Violin plots for the distribution of RMSE, MAE, and r^2 for each ML algorithm in the 1000 trials in the test set of H at top sites dataset.

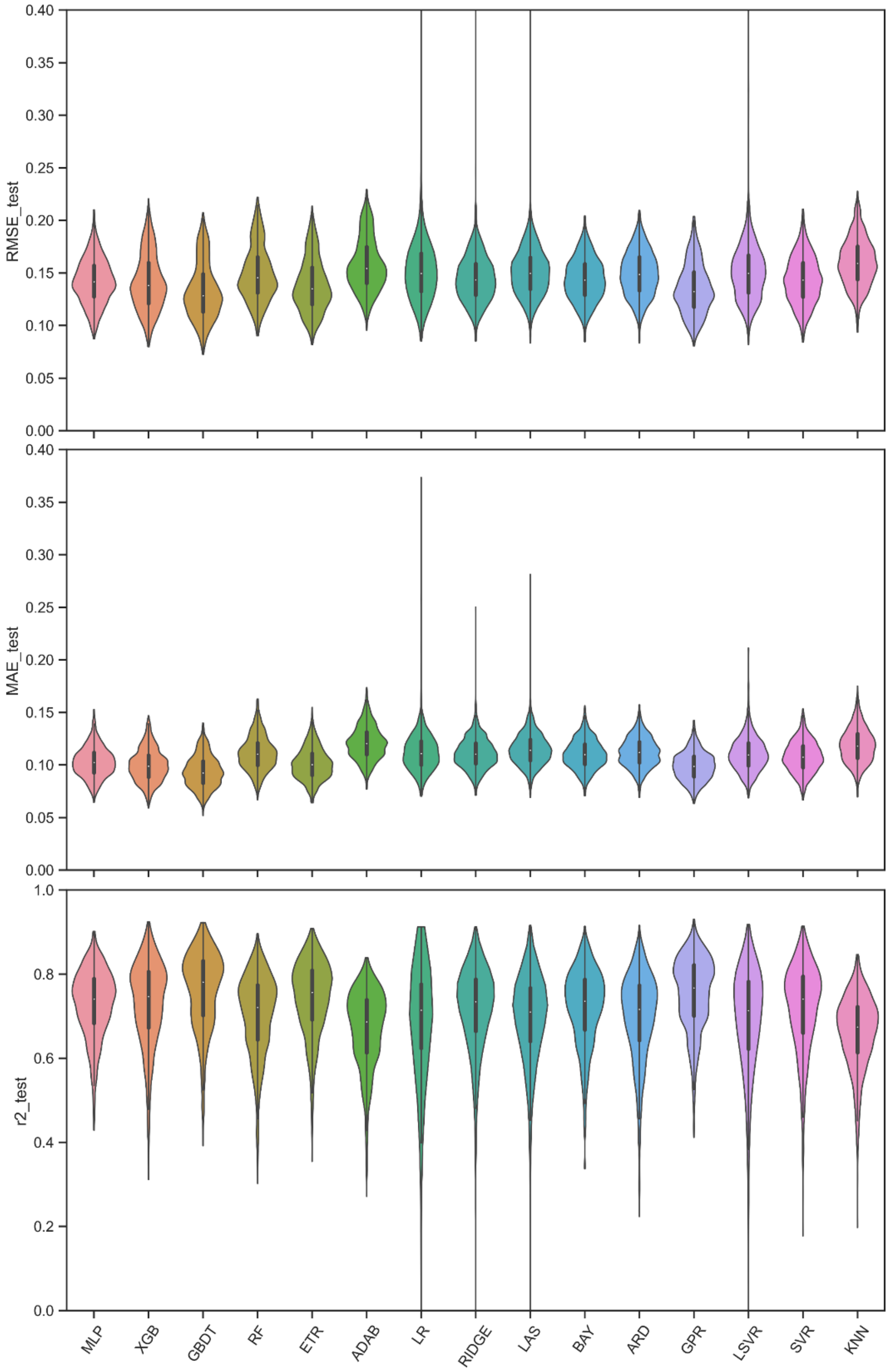

Supplementary Fig. 11 Violin plots for the distribution of RMSE, MAE, and r^2 for each ML algorithm in the 1000 trials in the test set of H at bridge sites dataset.

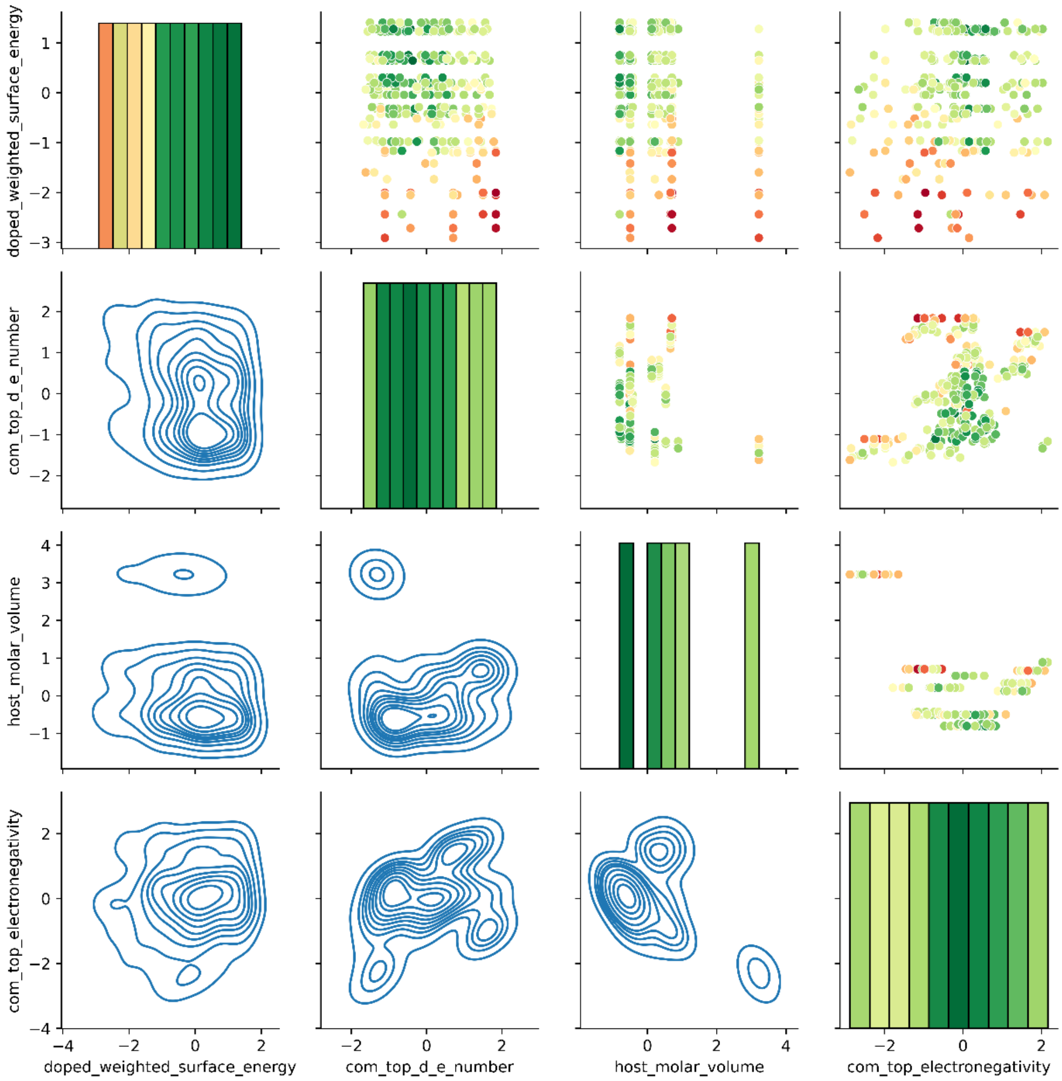

Supplementary Fig. 12 Distribution and variation trends of the C-H dissociation energy barrier for the H at top sites pathway with respect to doped_weighted_surface_energy, com_top_d_e_number, host_molar_volume, and com_top_electronegativity.

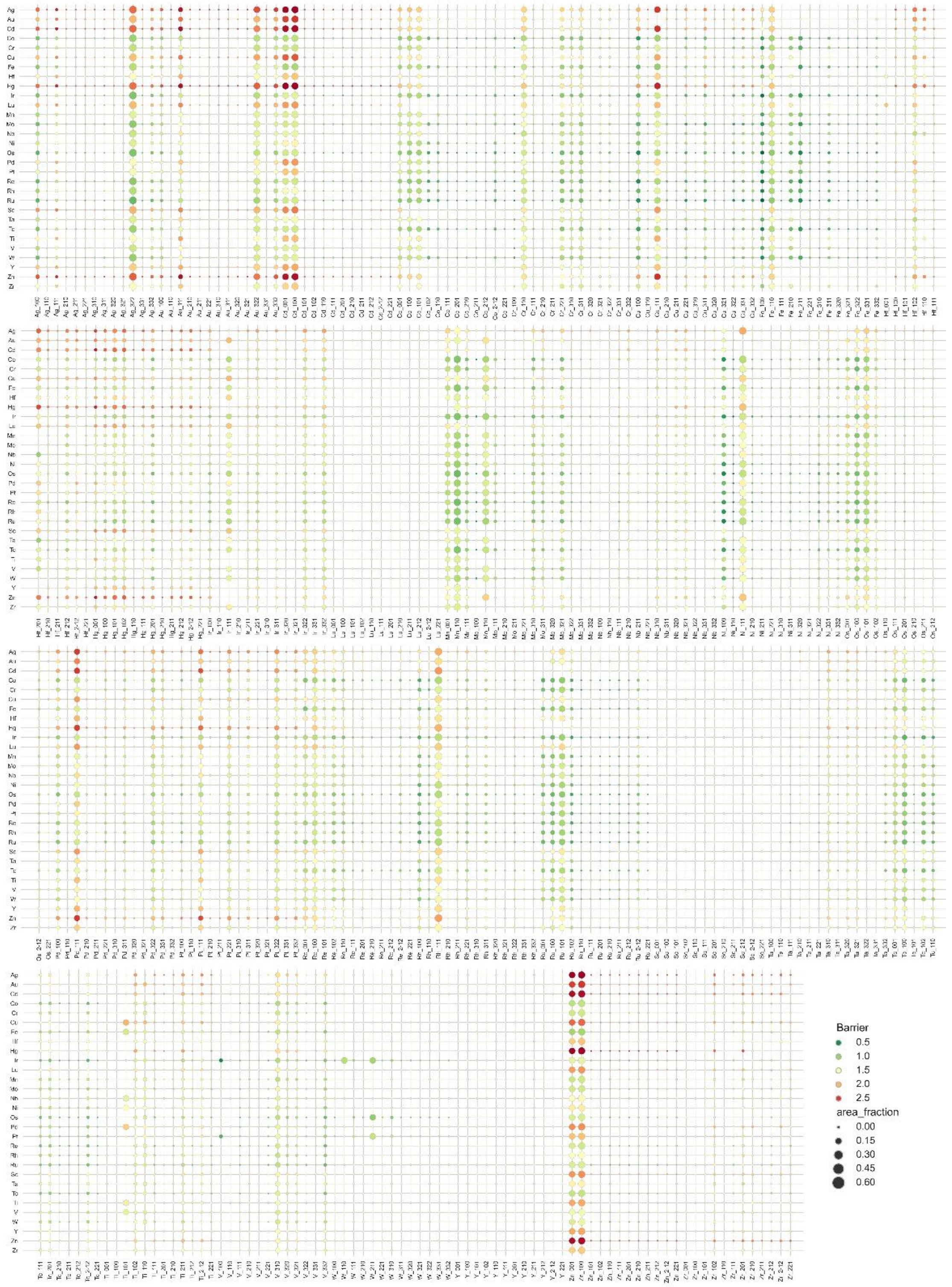

Supplementary Fig. 13 Heat map of C-H dissociation energy barrier predicted by GBDT model for H at the top sites.

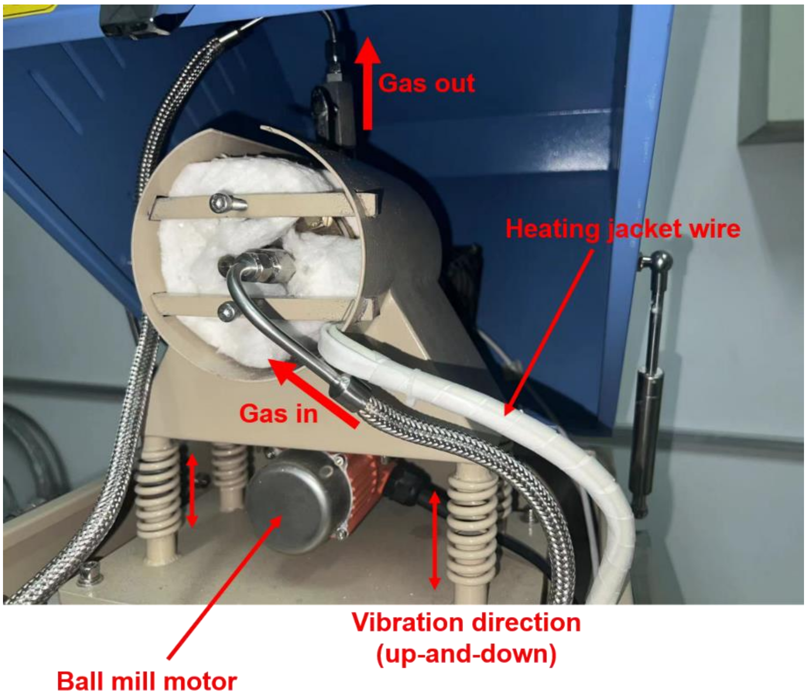

Supplementary Fig. 14 The schematic of the grinder and the movement manner (up-and-down) of the reactor under vibration mode.

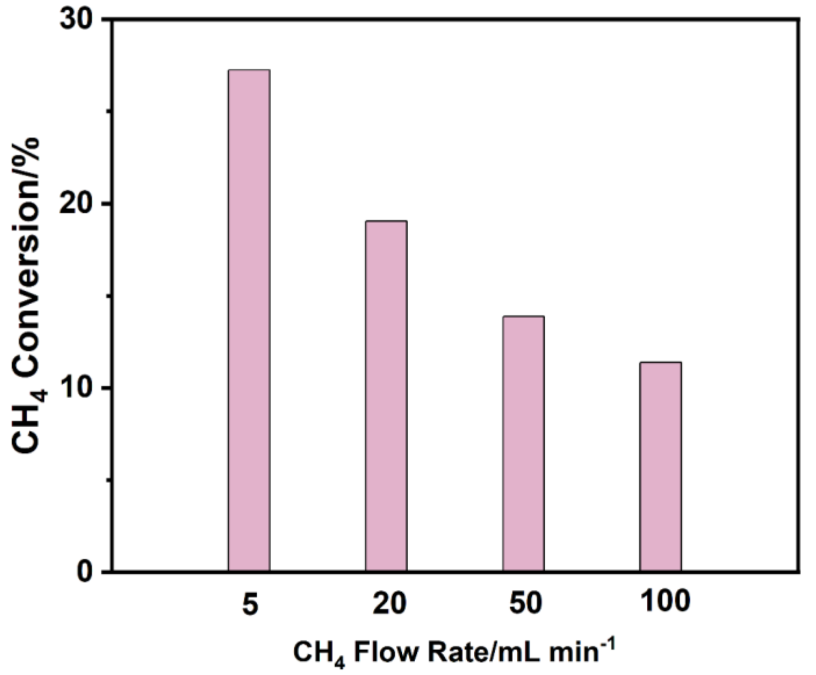

Supplementary Fig. 15 CH_4 conversion on Ir/Ni under different CH_4 flow rates

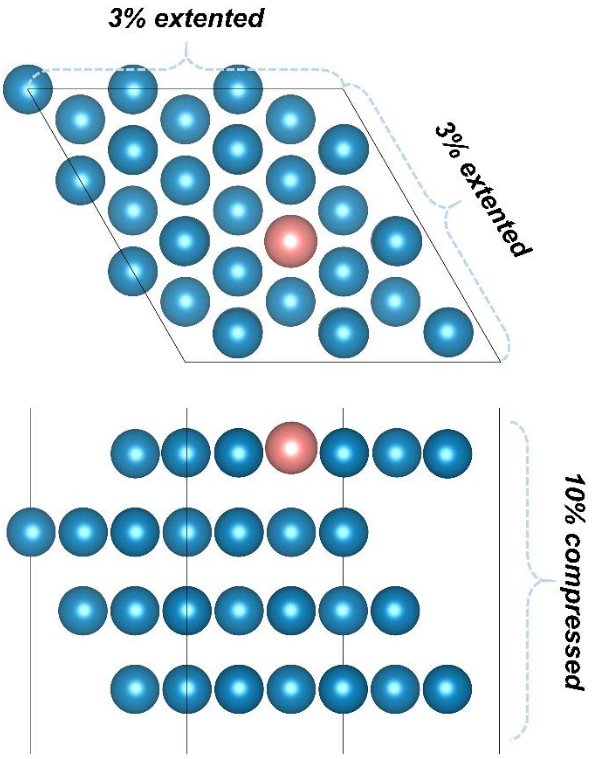

Supplementary Fig. 16 Collision deformation model of the Ni(111) surface

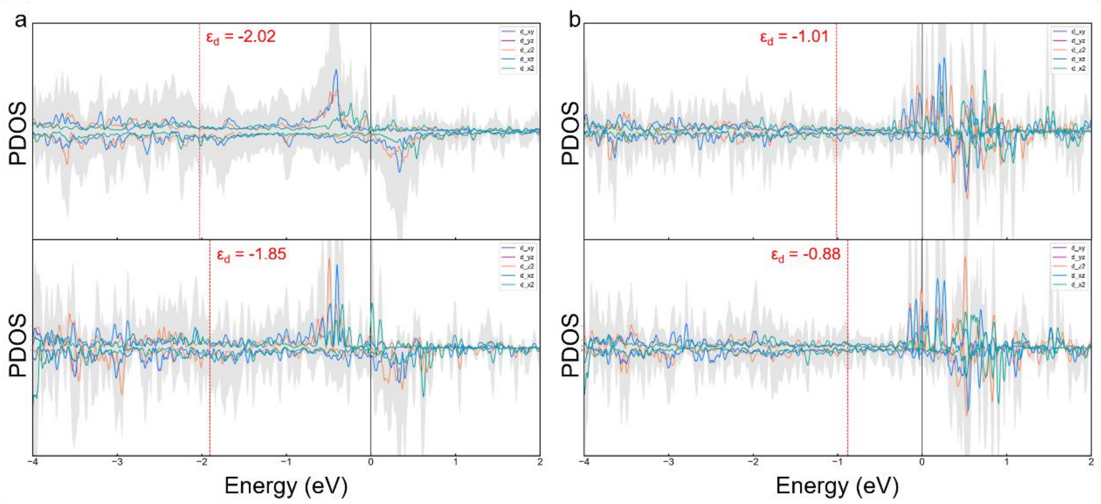

Supplementary Fig. 17 a, The projected density of states (PDOS) of Ir in Ir/Ni(111) before (top) and after (bottom) collision deformation. **b**, The PDOS of Re in Re/Ni(111) before (top) and after (bottom) collision deformation.

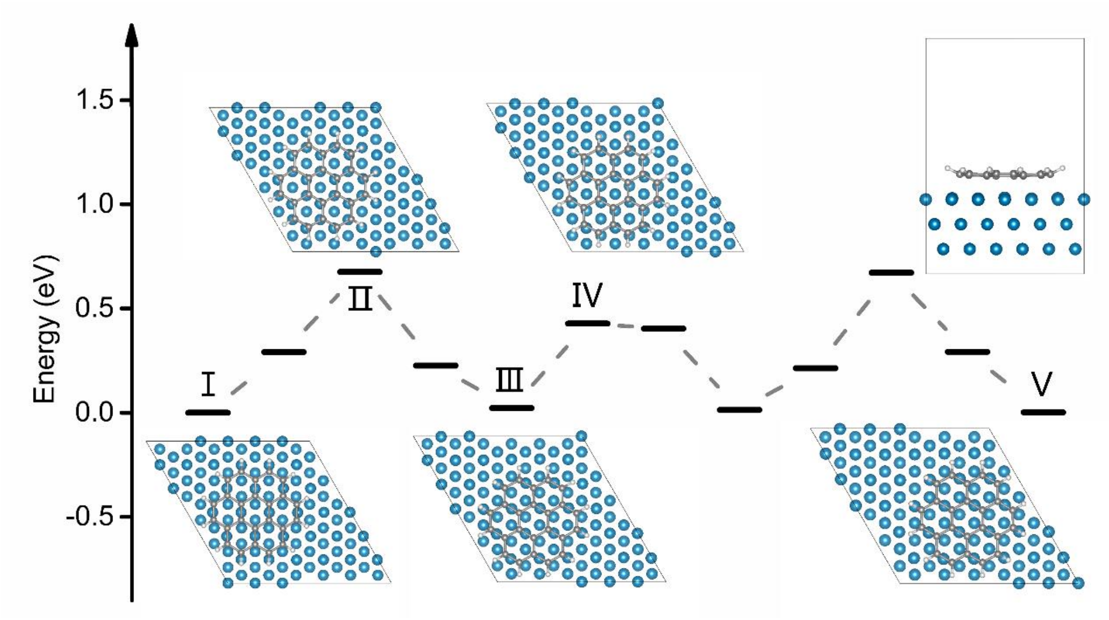

Supplementary Fig. 18 Reaction energy diagram and configurations of intermediates of graphene slip-on pure Ni (111) surface..

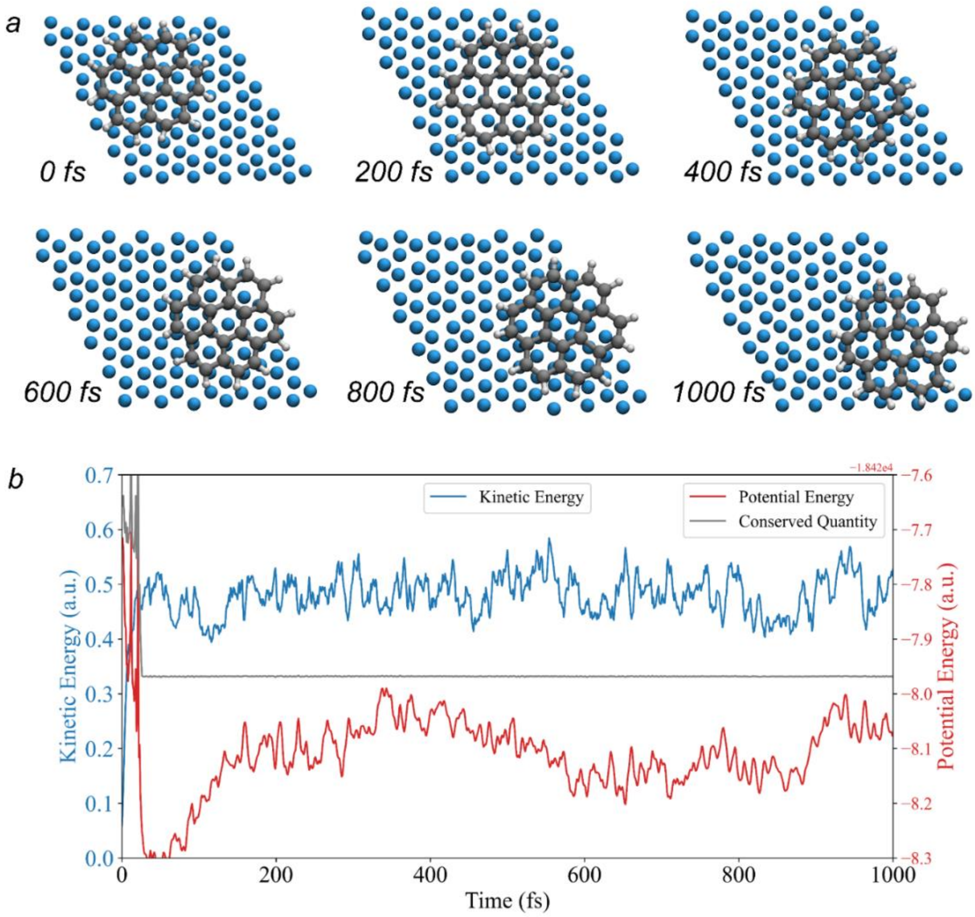

Supplementary Fig. 19 Snapshot (a) and energy (b) of carbon slip on Ni (111) surface after initial velocity of 0.001 Å/fs is applied.

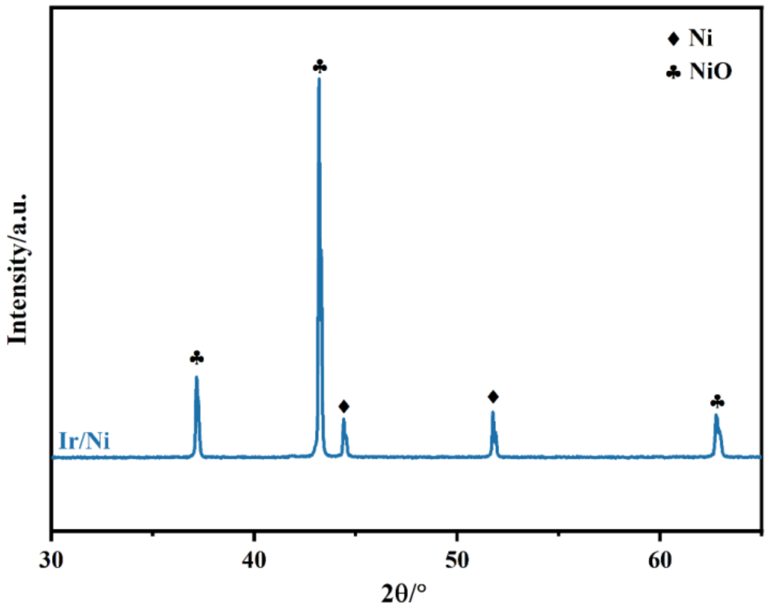

Supplementary Fig. 20 X-ray diffraction (XRD) profiles of Ir/Ni SAA

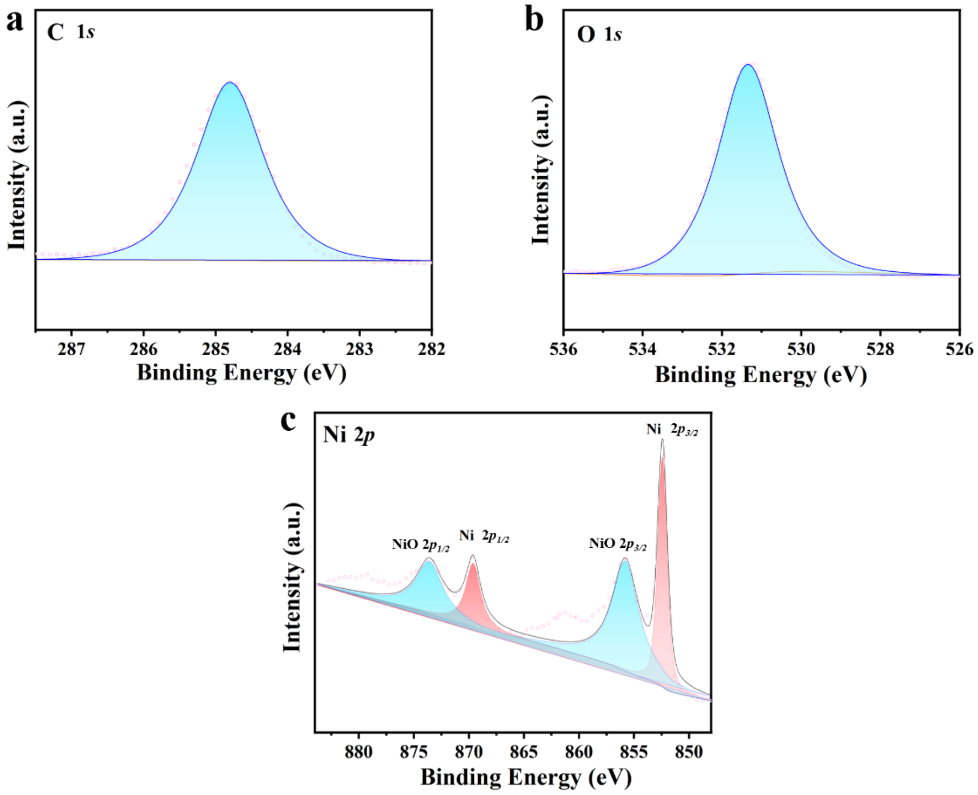

Supplementary Fig. 21 The X-ray photoelectron spectroscopy (XPS) spectra of Ni 2*p*, O 1*s* and C 1*s* over Re/Ni SAA.

Due to the high dispersion of Re and its low loading, XPS was unable to detect the signal of Re.

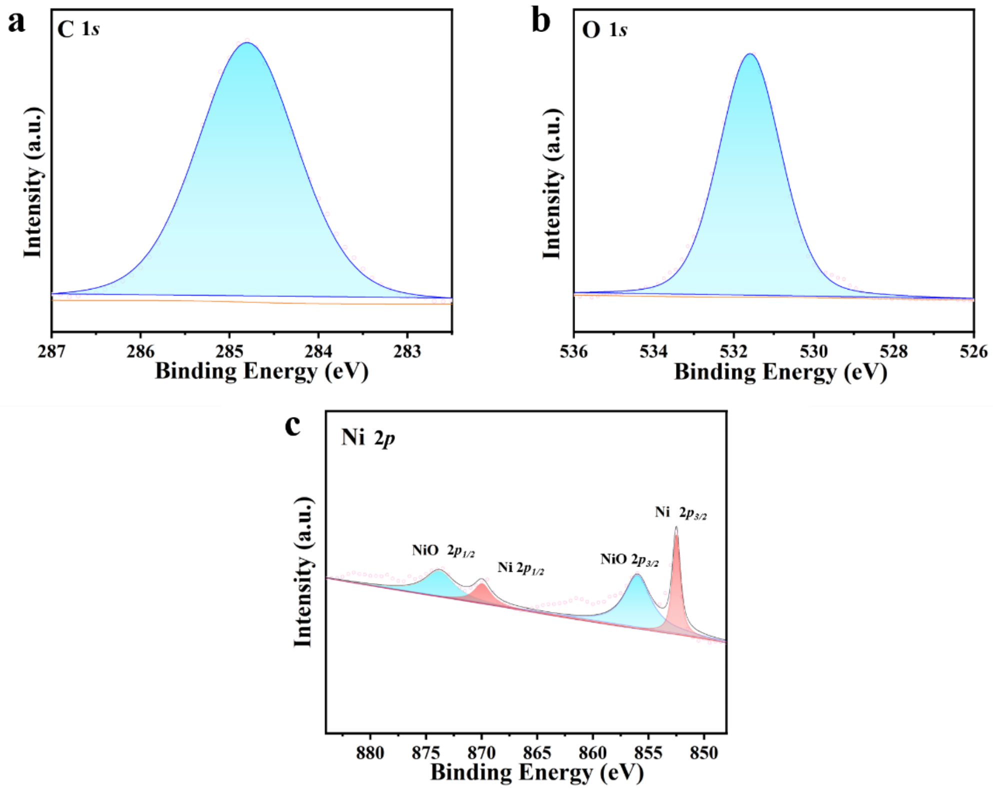

Supplementary Fig. 22 The XPS spectra of Ni 2*p*, O 1*s* and C 1*s* over Ir/Ni SAA

Due to the high dispersion of Ir and its low loading, XPS was unable to detect the signal of Ir.

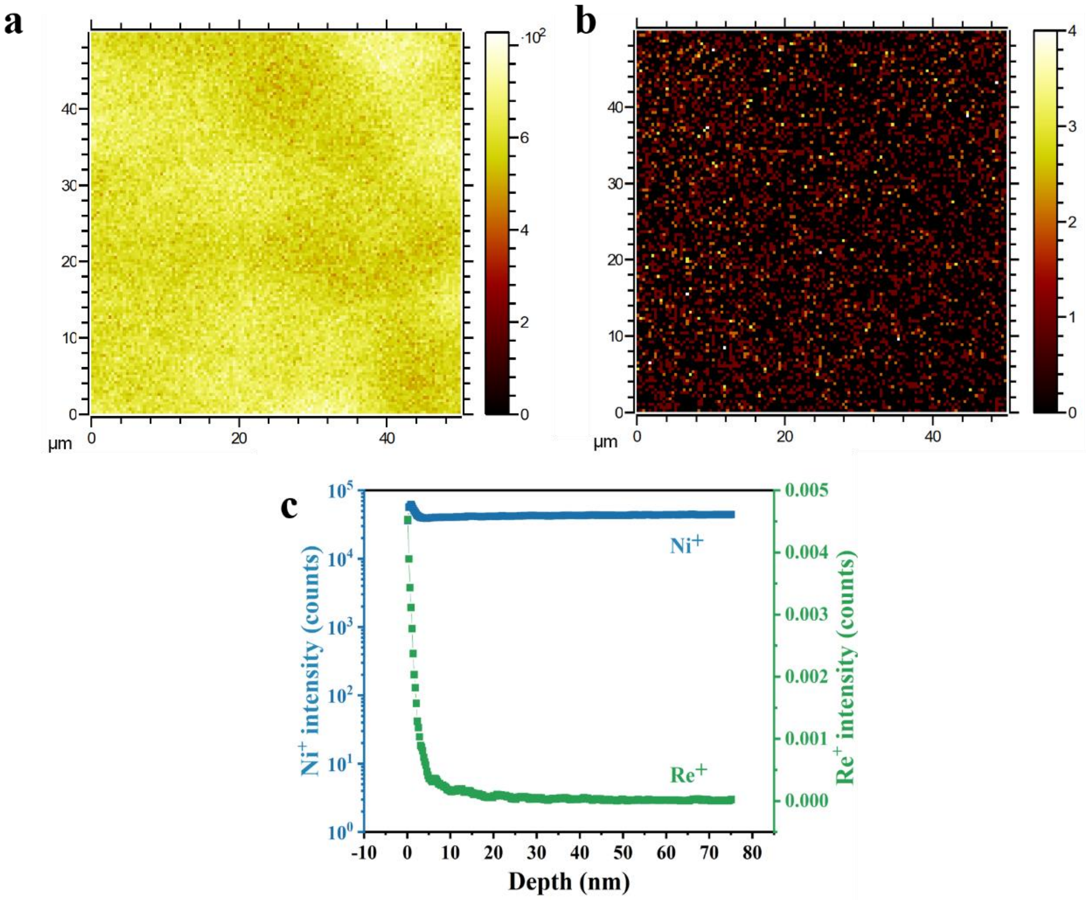

Supplementary Fig. 23 Abundance maps of (a) Ni^+ and (b) Re^+ over a 50 × 50 μm^2 field of view from the Re/Ni surface region. (c) The Ni^+ and Re^+ signals were obtained by time of flight secondary ion mass spectrometry (TOF-SIMS) analysis with respect to the diffusion depth.

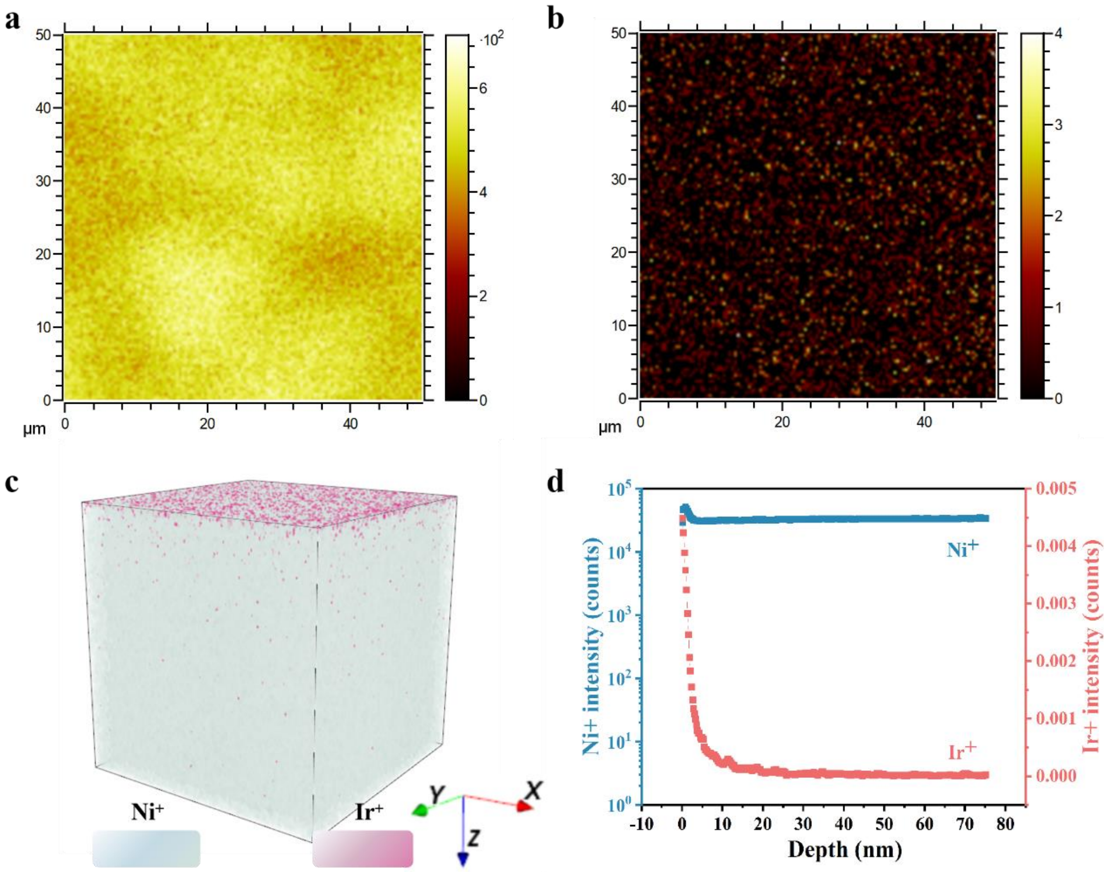

Supplementary Fig. 24 Abundance maps of (a) Ni^+ and (b) Ir^+ over a 50 × 50 μm^2 field of view from the Ir/Ni surface region. (c) The 3D TOF-SIMS map of Ir signal on the Ni substrate. (d) The Ni^+ and Ir^+ signals were obtained by TOF-SIMS analysis with respect to the diffusion depth.

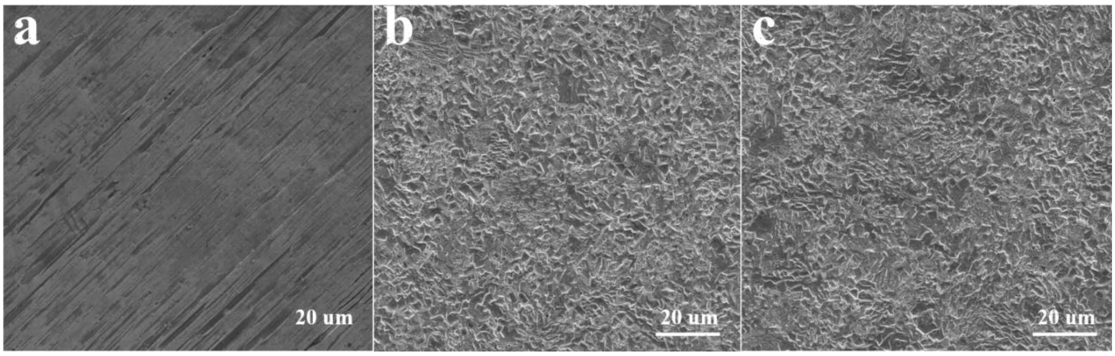

Supplementary Fig. 25 Scanning electron microscopy (SEM) images of Ni(a), Re/Ni(b) and Ir/Ni(c) metal surface.

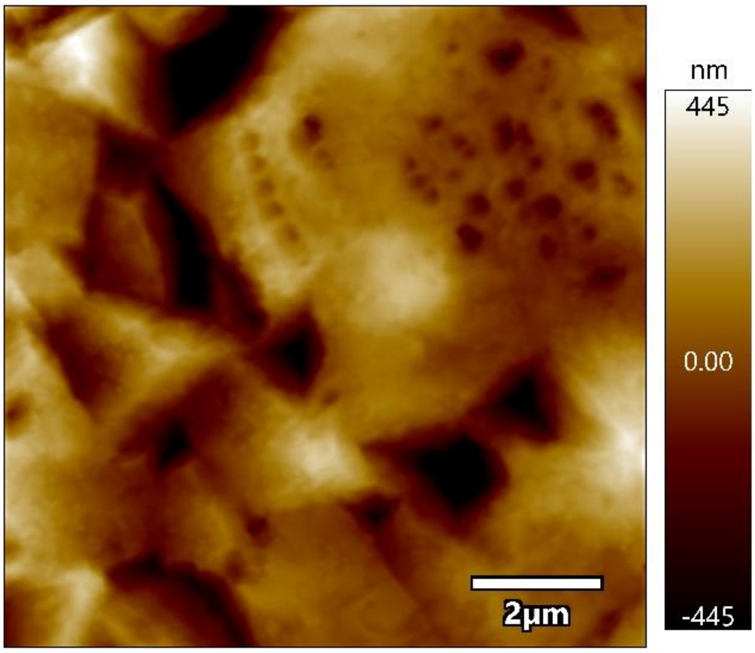

Supplementary Fig. 26 The 2D surface geometry images of the Ir/Ni sample from atomic force microscope (AFM).

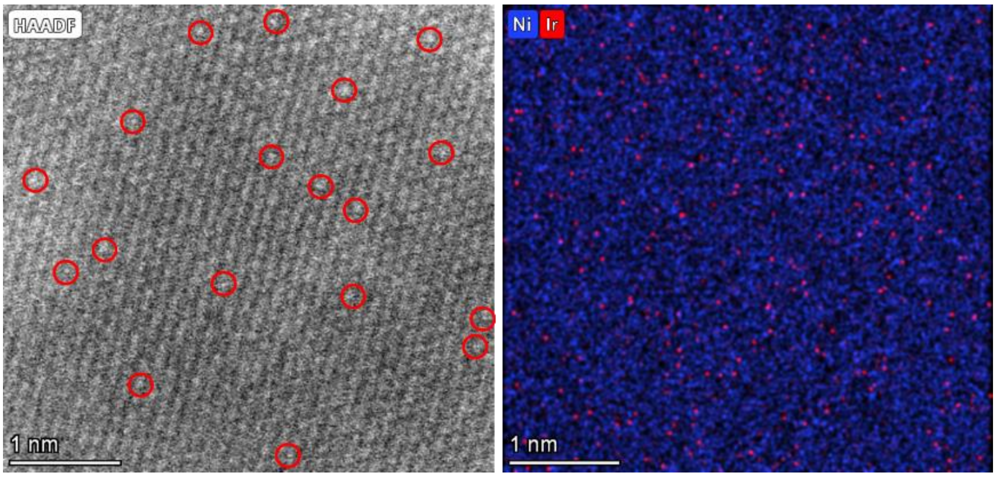

Supplementary Fig. 27 HAADF-STEM image and elemental mapping of Ir/Ni.

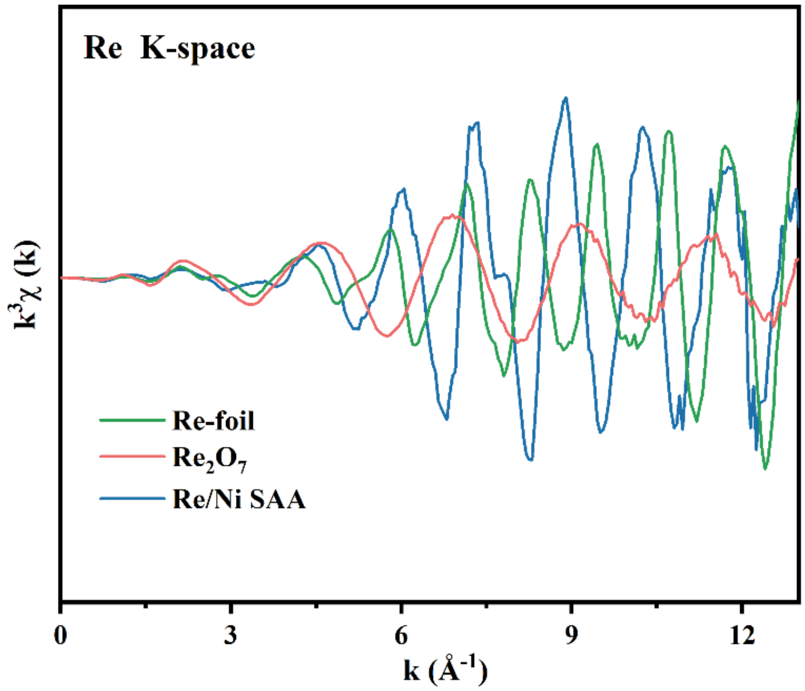

Supplementary Fig. 28 k^3-Weighted EXAFS spectra in k-space of Re/Ni catalyst with Re foil and Re_2O_7 as references.

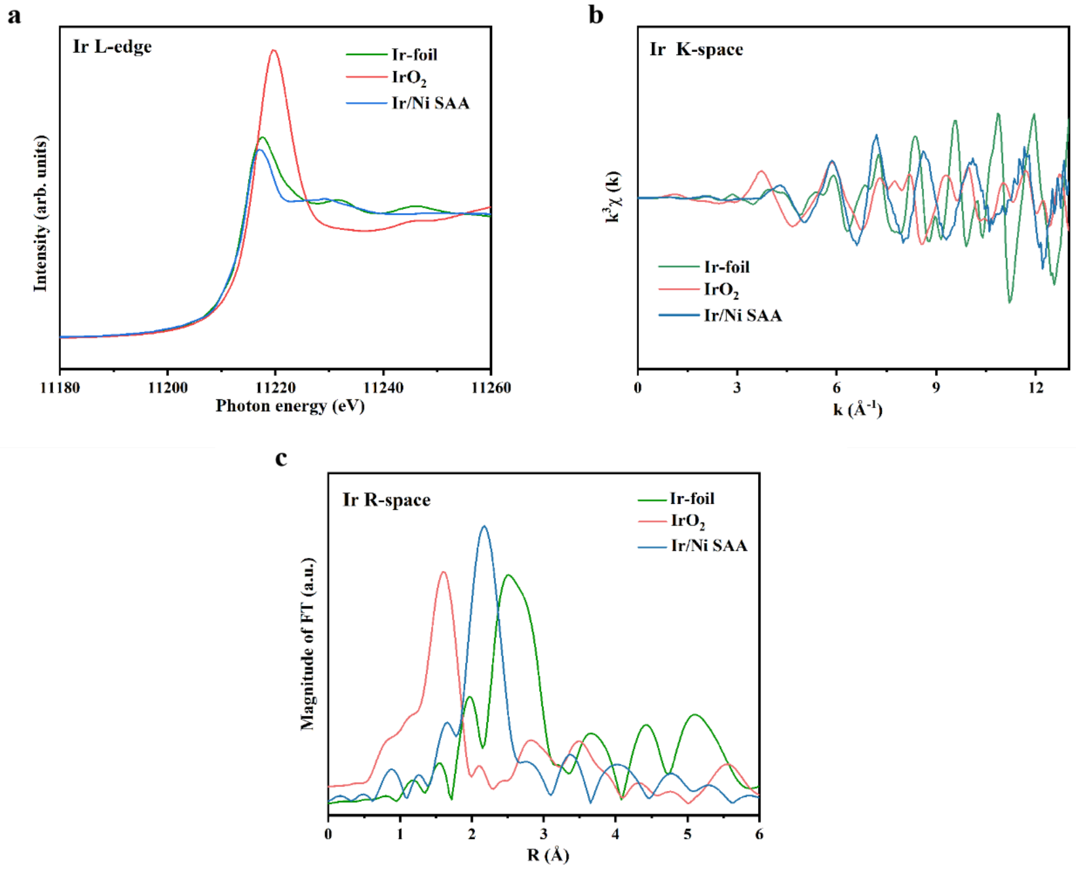

Supplementary Fig. 29 a X-ray absorption near-edge spectroscopy (XANES) spectra of Ir L_3-edge

from Ir/Ni catalyst with Ir foil and IrO_2 as references. b Ir L_3-edge EXAFS in R space from Ir/Ni catalyst with Ir foil and IrO_2 as references. c k^3-Weighted EXAFS spectra in k-space of Ir/Ni catalyst with Ir foil and IrO_2 as references.

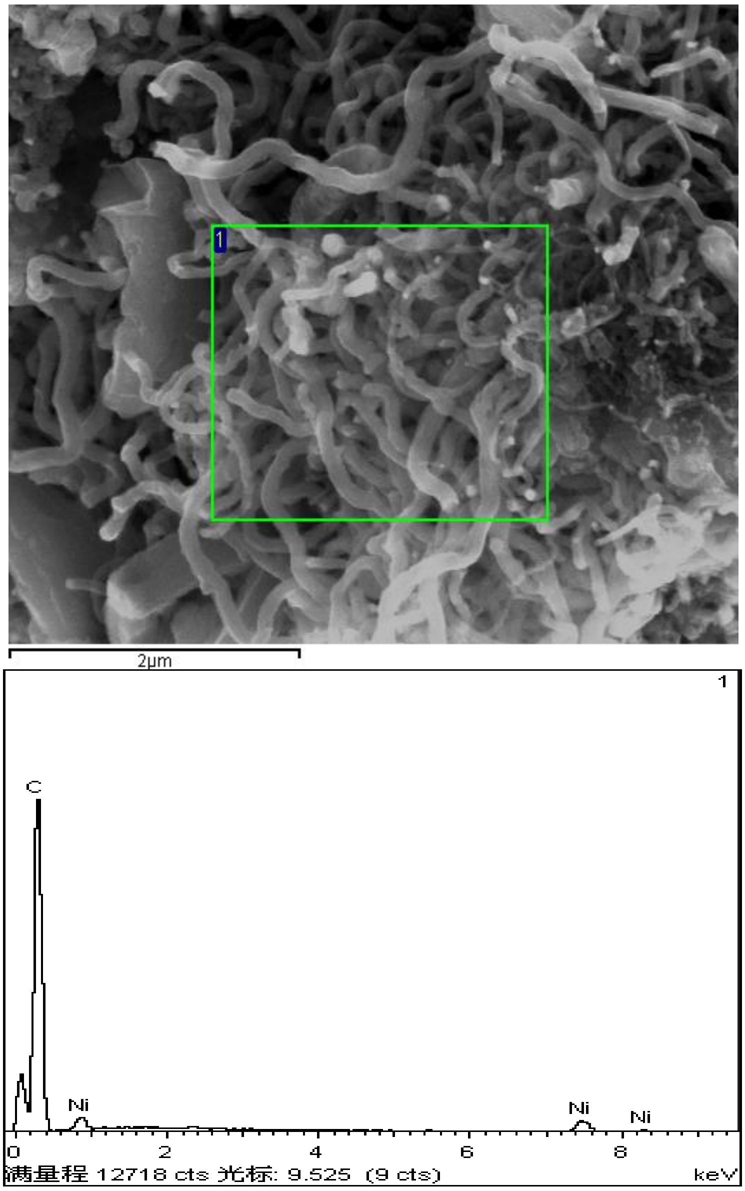

Supplementary Fig. 30 The SEM images of the byproduct powder sample and the energy dispersive spectroscopy (EDS) results of the area labeled by a green rectangle

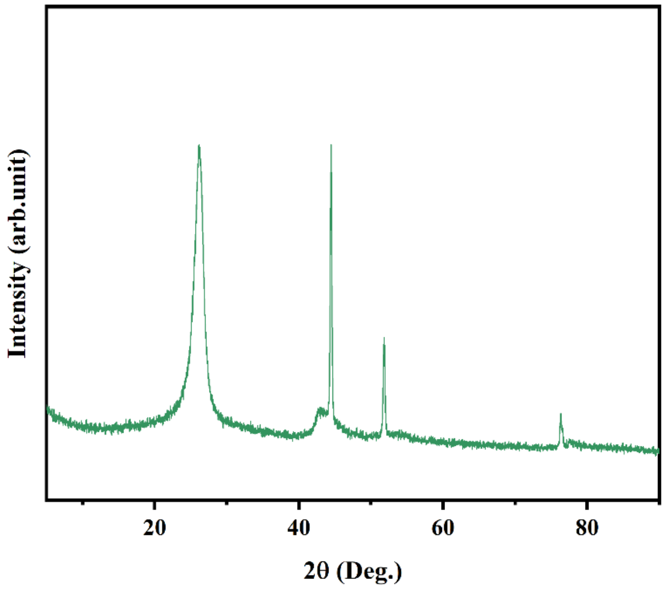

Supplementary Fig. 31 The XRD spectra for carbon products

The peak around 26° was assigned to the amorphous carbon species, and the residual peaks at 44.5°, 51.8° and 76.4° were ascribed to metal Ni.

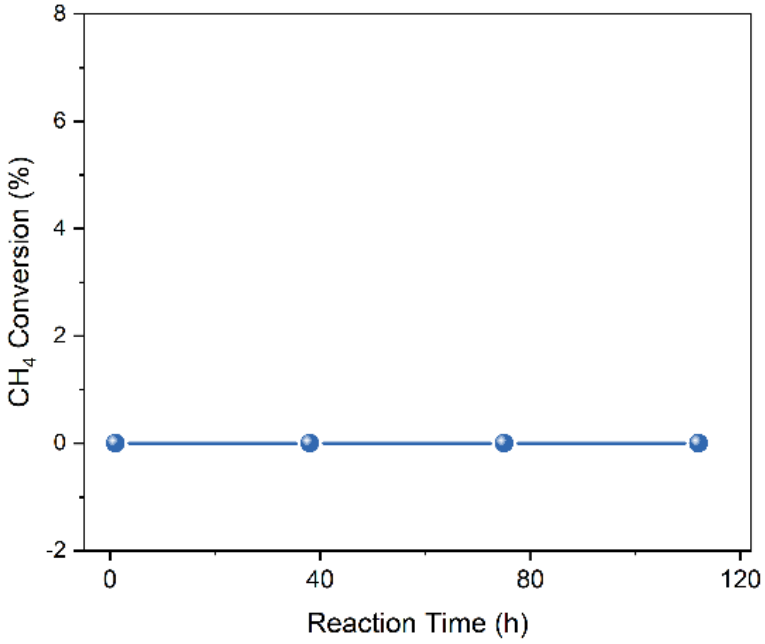

Supplementary Fig. 32 CH_4 conversion of carbon powder

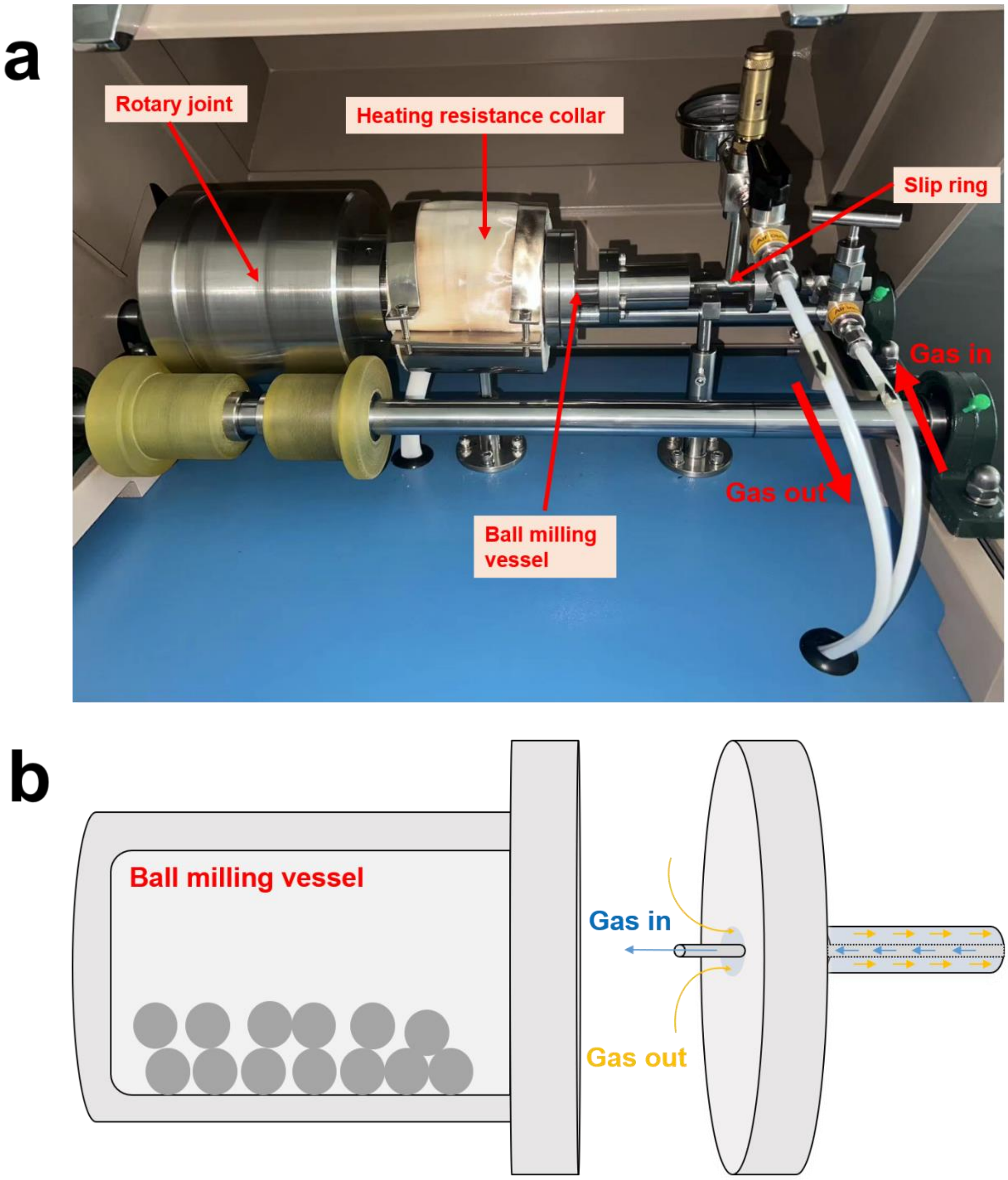

Supplementary Fig. 33 (a) Schematic diagram of self-made rolling ball mill. (b) Schematic diagram of ball milling container and gas flow system.

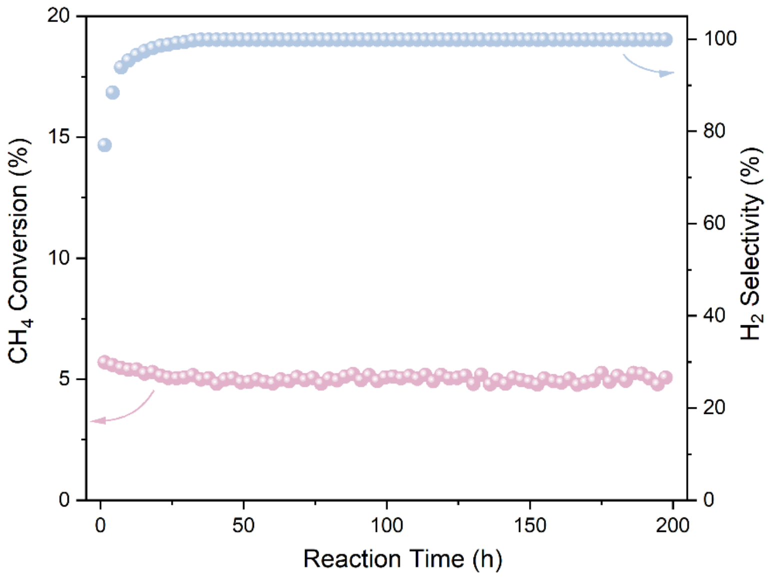

Supplementary Fig. 34 Methane cracking activity of Ir/Ni in rolling ball mill.

The activity of Ir/Ni in rotating reactor was lower than that in vibrating reactor, which may be due to:

(1) The smaller inner diameter of rotating reactor resulted in weaker collision force than that for vibrating reactor.

(2) For the structure of rotating reactor, the gaseous inlet and outlet were nearby, which might affect the gaseous flow route and inhibit the gaseous products replacement in rotating reactor.

4. Supplementary References

1. Jain, A. *et al.* Commentary: The Materials Project: A materials genome approach to accelerating materials innovation. *APL Mater.* **1**, 011002 (2013).
2. Sun, W. & Ceder, G. Efficient creation and convergence of surface slabs. *Surf. Sci.* **617**, 53–59 (2013).
3. Guyon, I., Weston, J. & Barnhill, S. Gene Selection for Cancer Classification using Support Vector Machines.
4. Pedregosa, F. *et al.* Scikit-learn: Machine Learning in Python. *Mach. Learn. PYTHON*.
5. He, K., Zhang, X., Ren, S. & Sun, J. Delving Deep into Rectifiers: Surpassing Human-Level Performance on ImageNet Classification. Preprint at http://arxiv.org/abs/1502.01852 (2015).
6. Chen, T. & Guestrin, C. XGBoost: A Scalable Tree Boosting System. in *Proceedings of the 22nd ACM SIGKDD International Conference on Knowledge Discovery and Data Mining* 785–794 (2016). doi:10.1145/2939672.2939785.
7. Wang, G., Jin, Y., Liu, G. & Li, Y. Production of Hydrogen and Nanocarbon from Catalytic Decomposition of Methane over a Ni–Fe/Al_2O_3 Catalyst. *Energy Fuels* **27**, 4448–4456 (2013).
8. Bayat, N., Rezaei, M. & Meshkani, F. Hydrogen and carbon nanofibers synthesis by methane decomposition over Ni–Pd/Al_2O_3 catalyst. *Int. J. Hydrog. Energy* **41**, 5494–5503 (2016).
9. Al-Fatesh, A. S. *et al.* Production of hydrogen by catalytic methane decomposition over alumina supported mono-, bi- and tri-metallic catalysts. *Int. J. Hydrog. Energy* **41**, 22932–22940 (2016).
10. Pudukudy, M., Yaakob, Z. & Akmal, Z. S. Direct decomposition of methane over SBA-15 supported Ni, Co and Fe based bimetallic catalysts. *Appl. Surf. Sci.* **330**, 418–430 (2015).
11. Upham, D. C. *et al.* Catalytic molten metals for the direct conversion of methane to hydrogen and separable carbon. *Science* **358**, 917–921 (2017).
12. Shen, Y. & Lua, A. C. Synthesis of Ni and Ni–Cu supported on carbon nanotubes for hydrogen and carbon production by catalytic decomposition of methane. *Appl. Catal. B Environ.* **164**, 61–69 (2015).
13. Ashok, J., Reddy, P. S., Raju, G., Subrahmanyam, M. & Venugopal, A. Catalytic Decomposition of Methane to Hydrogen and Carbon Nanofibers over Ni−Cu−SiO_2 Catalysts. *Energy Fuels* **23**, 5–13 (2009).
14. Suelves, I., Pinilla, J. L., Lázaro, M. J., Moliner, R. & Palacios, J. M. Effects of reaction conditions

on hydrogen production and carbon nanofiber properties generated by methane decomposition in a fixed bed reactor using a NiCuAl catalyst. *J. Power Sources* **192**, 35–42 (2009).

15. Chen, L. *et al.* Ternary NiMo-Bi liquid alloy catalyst for efficient hydrogen production from methane pyrolysis. *Science* **381**, 857-861(2023).

16. Lua, A. C. & Wang, H. Y. Hydrogen production by catalytic decomposition of methane over Ni-Cu-Co alloy particles. *Appl. Catal. B Environ.* **156–157**, 84–93 (2014).
