# Supplementary material for: Machine learning aided design of single-atom alloy catalysts for methane cracking
Source: Nat Commun. 2024 Jul 18;15:6036. doi: 10.1038/s41467-024-50417-7 (PMC11255339; doi:10.1038/s41467-024-50417-7)
Supplement: Supplementary file 3 — Description of Additional Supplementary Files [file 41467_2024_50417_MOESM3_ESM.pdf]

Description of Additional Supplementary Files

Supplementary Movie 1: The generated carbon fragment could easily slip from the Ni (111) surface.
