# Supplementary figures and images for: Machine learning aided design of single-atom alloy catalysts for methane cracking
Source: Nat Commun. 2024 Jul 18;15:6036. doi: 10.1038/s41467-024-50417-7 (PMC11255339; doi:10.1038/s41467-024-50417-7)

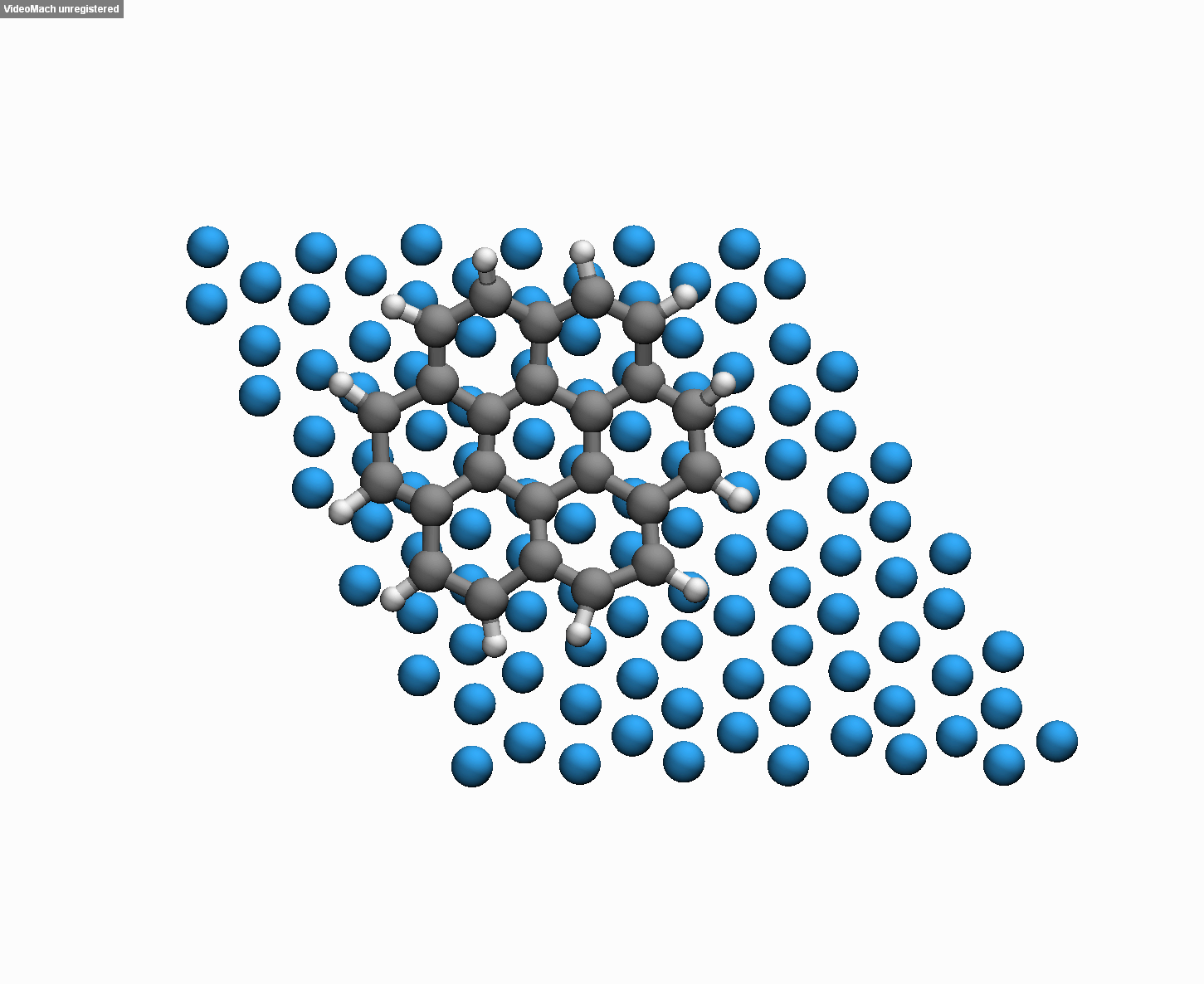

Supplement: Supplementary file 4 — Supplementary Movie 1 [file 41467_2024_50417_MOESM4_ESM.gif]
